# Supplementary material for: Replisomes restrict SMC translocation in vivo
Source: Nat Commun. 2025 Aug 4;16:7151. doi: 10.1038/s41467-025-62596-y (PMC12322038; doi:10.1038/s41467-025-62596-y)
Supplement: Supplementary file 1 — Supplementary Information [file 41467_2025_62596_MOESM1_ESM.pdf]

Supplementary Information For

Replisomes restrict SMC translocation *in vivo*

Qin Liao, Hugo B. Brandão, Zhongqing Ren, and Xindan Wang

Inventory of Supplementary Information:

11 Supplementary Figures and Figure Legends

- **Supplementary Fig. 1:** Determining replisome dynamics by simulating experimental MFA data. Related to Fig. 1.
- **Supplementary Fig. 2:** SMC enrichment upon ParB expression in a strain containing a single *parS* at -59° (BWX5297). Related to Fig. 1.
- **Supplementary Fig. 3:** Illustrations of Hi-C features. Related to Fig. 2.
- **Supplementary Fig. 4:** HPUra does not affect SMC translocation rate. Related to Figs. 3 and 4.
- **Supplementary Fig. 5:** Measuring the delay of SMC before bypassing the replisome in the collisions. Related to Figs. 4 and 5.
- **Supplementary Fig. 6:** Calibration of SMC spontaneous disassociation rate at 42°C. Related to Fig. 6.
- **Supplementary Fig. 7:** Sweeping of bypassing rates and unloading rates in the one-sided head-to-tail collision. Related to Fig. 6a-e.
- **Supplementary Fig. 8:** Sweeping of bypassing rates and unloading rates in the head-on collision. Related to Fig. 6f-j.
- **Supplementary Fig. 9:** Moving replisomes have a stronger effect on SMC translocation than stalled replisomes. Related to Figs. 5 and 7.
- **Supplementary Fig. 10:** SMC was further slowed down by moving replisomes in the head-to-tail collision. Related to Fig. 7.
- **Supplementary Fig. 11:** Simulations to generate arc 2 on Hi-C maps. Related to Figs. 2 and 7.

Supplementary Table 1: Bacterial strains, plasmids and oligonucleotides used in this study.

Supplementary Methods

Supplementary References: This includes the references cited in the Supplementary Information.

Supplementary Figure 1

a

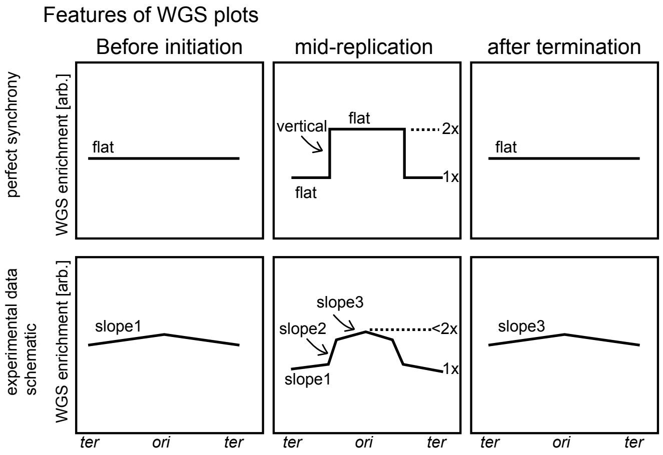

b

Parameters	Values	Units	Fit/Fixed
DNAP_load_time_constant	5	1/min	fit
low_temp_DNAP_rate	22	kb/min	fit
high_temp_DNAP_rate	66.0	kb/min	fit
DNAP_stall_time_constant	44	1/min	fit
fraction_pre_existing_DNAP	0.18	N/A	fit
num_chromosomes_for_fit	11800	N/A	fixed
bin_size_for_fit	10	kb	fixed
chromosome_length_in_bins	404	kb	fixed
time_for_DNAP_loading	15	min	fixed
time_at_low_temperature	15	min	fixed

c

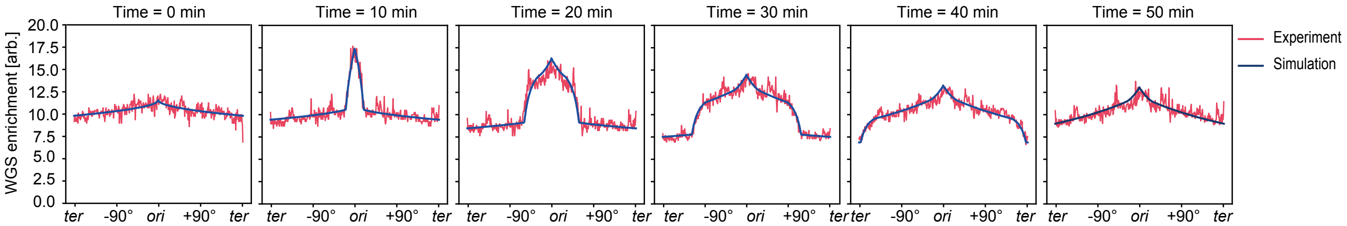

d

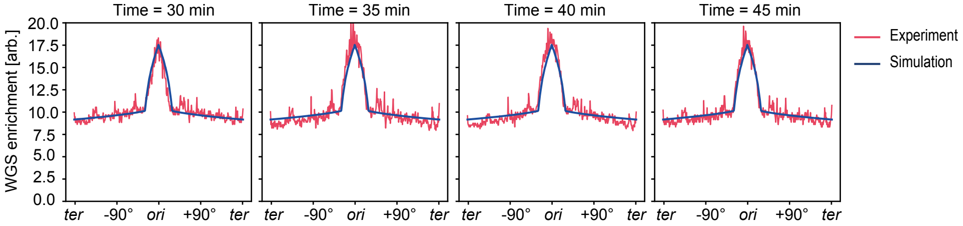

e

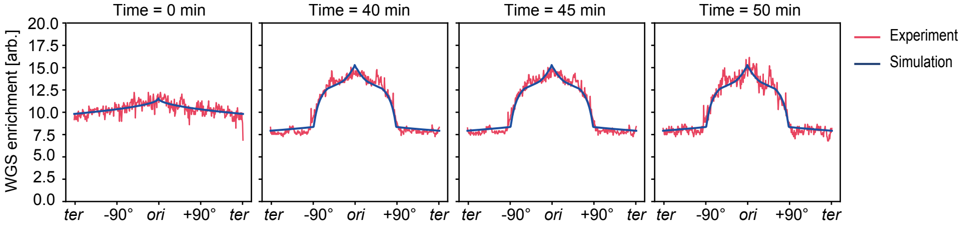

f

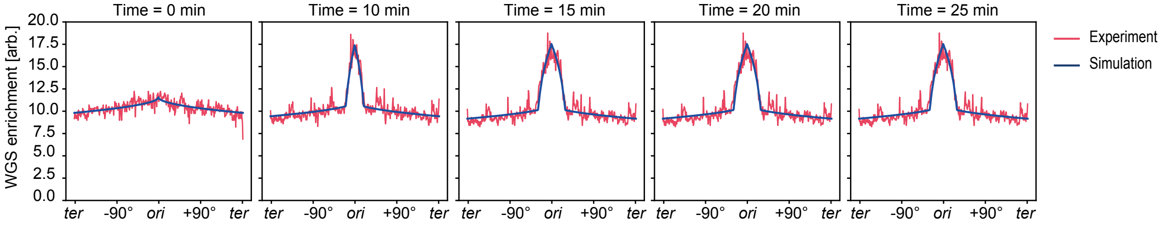

Supplementary Fig. 1: Determining replisome dynamics by simulating experimental MFA data. Related to Fig. 1.
(a) **I**llustrations depicting features of perfect replication synchrony (top) and the MFA plots in this study.
(b) Parameters that produced the best fit for MFA plots (see c-f below and Supplementary Methods under "calibration of replisome dynamics model" for details)
(c) Comparison of simulated replication profiles (blue curves) with experimental MFA plots (red curves) for data in Fig. 1c.
(d-f) Comparison of simulated replication profiles (blue curves) with experimental MFA plots (red curves) for experiments presented in Figs. 4b, 5b and 5c, respectively.

Supplementary Figure 2

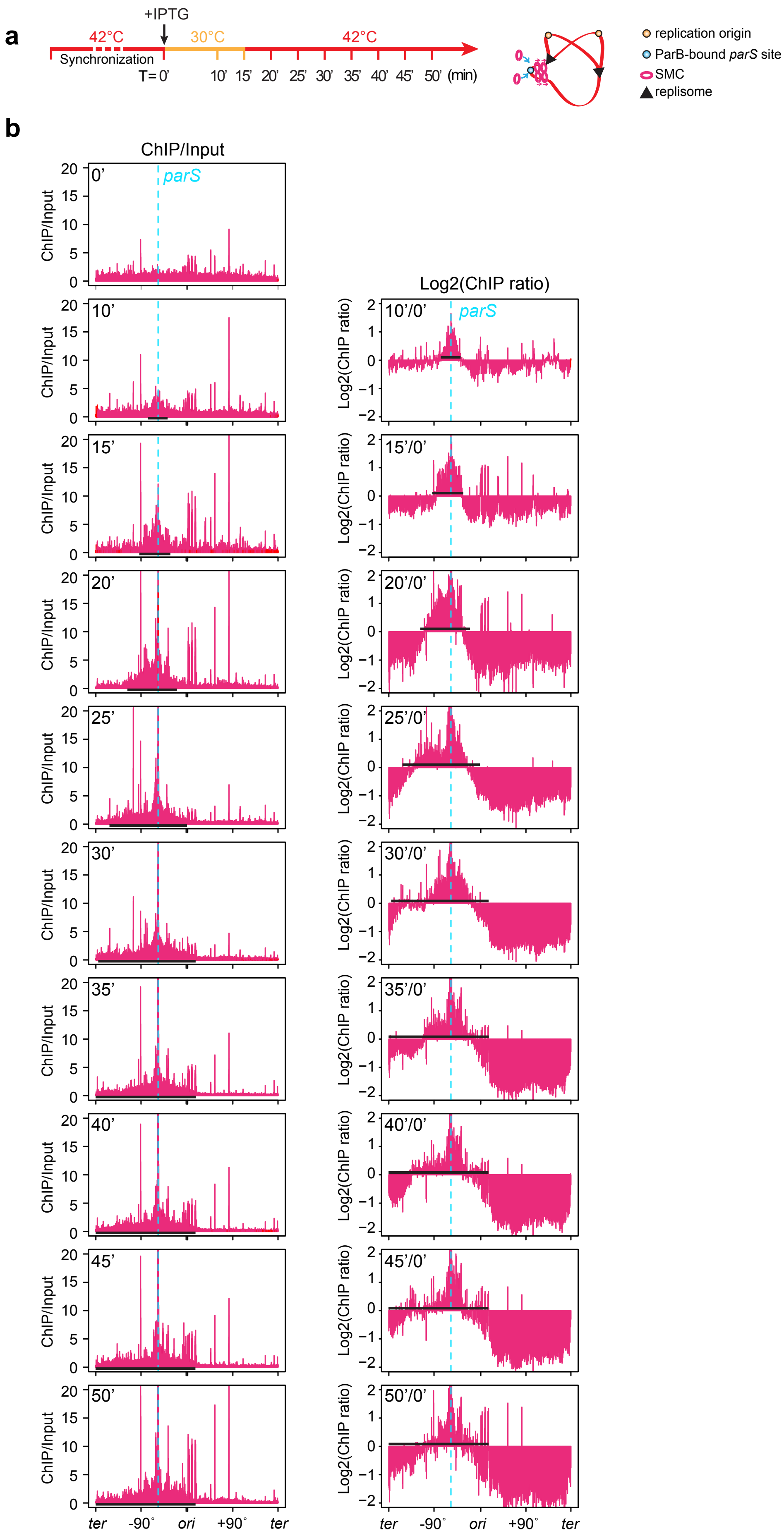

Supplementary Fig. 2: SMC enrichment upon ParB expression in a strain containing a single *parS* at -59° (BWX5297). Related to Fig. 1.
(a) Experimental timeline as shown in Fig. 1c.
(b) SMC ChIP-seq enrichment profiles of the samples used in Fig. 1c. anti-SMC ChIP enrichments (ChIP/input) were plotted in 1-kb bins (left panel). The ratio of ChIP enrichment at indicated time points relative to the onset of replication initiation (0') were plotted in log_2 scale in 5-kb bins (right panel). The *parS* site is indicated with cyan dashed lines. SMC enrichment zones are indicated as black bars in this figure, but as magenta bars in MFA plots in Fig. 1c.

Supplementary Figure 3

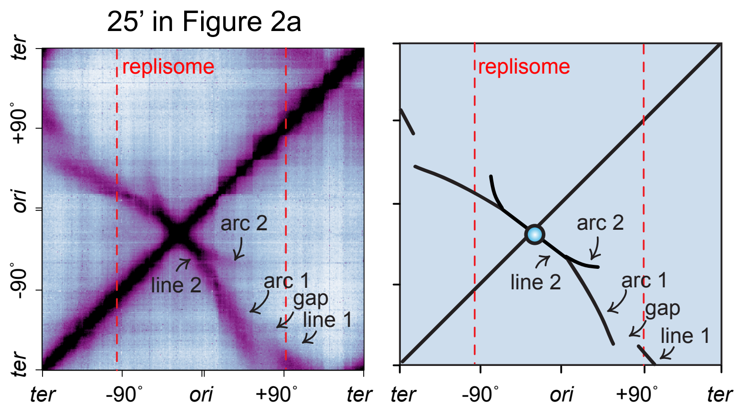

Supplementary Fig. 3: Illustrations of Hi-C features. Related to Fig. 2.
Left: Hi-C map shown in Fig. 2a (25'). Right: a schematic depicting major Hi-C features.

Supplementary Figure 4

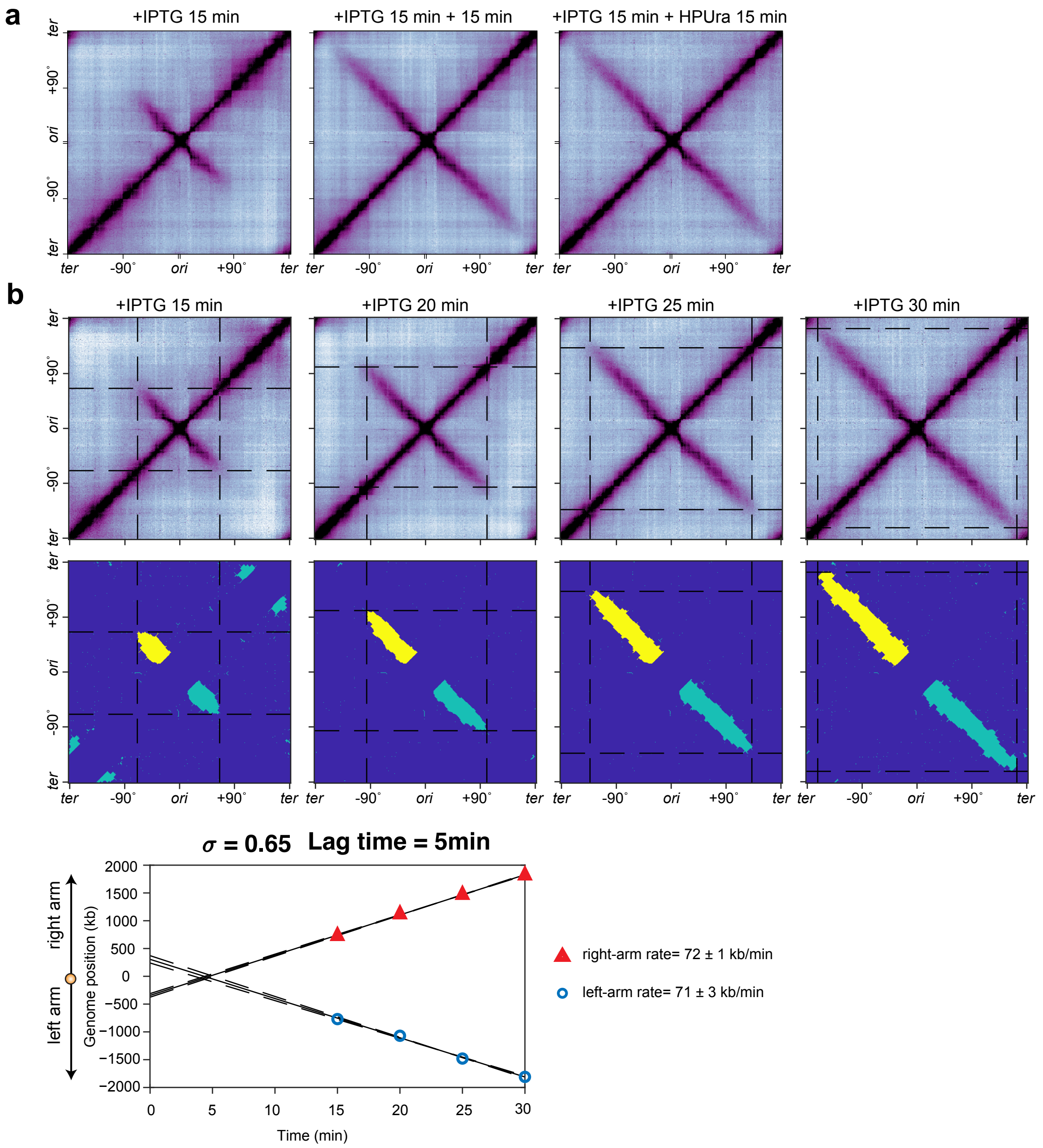

Supplementary Fig. 4: HPUra does not affect SMC translocation rate. Related to Figs. 3 and 4.
(a) Hi-C maps of a strain containing a single *parS* site at -1° and IPTG-inducible *parB* (BWX4310). Cells were arrested in G1. IPTG was added for 15 min to induce SMC loading, then cells were treated with or without HPUra for another 15 min. Without ongoing replication, SMC-mediated DNA zipping was not affected by HPUra.
(b) The DNA zipping rate at 42°C without SMC-replisome collisions was determined as described previously [1]. 0.65x standard deviation (s) above the averaged Hi-C contact score was used as the threshold. Top: time-course Hi-C maps shown in Fig. 4a. Middle: binary maps showing points with Hi-C contact scores higher than the threshold. Bottom: quantified DNA zipping at indicated time points. The SMC extrusion rate was calculated from the slope of the lines in the plot. Source data are provided as a Source Data file.

Supplementary Figure 5

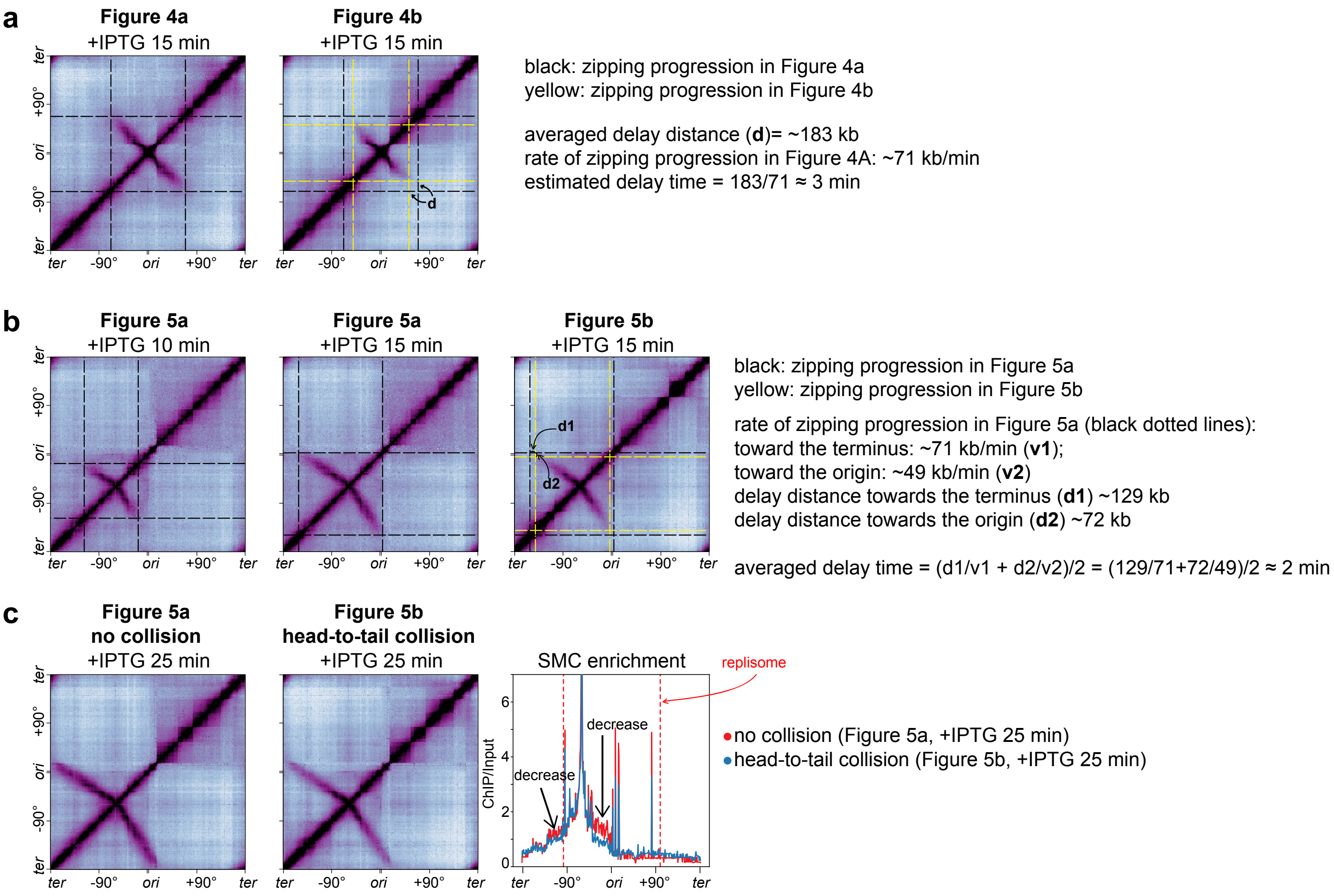

Supplementary Fig. 5: Measuring the delay of SMC before bypassing the replisome in the collisions. Related to Figs. 4 and 5.

(a) Experimental Hi-C maps for the control (no collision) and the two-sided head-to-tail collision at indicated time points shown in Fig. 4a and Fig. 4b, respectively. The strain contained a single *parS* site at -1°. DNA zipping progression in two conditions are indicated with black dashed lines and yellow dashed lines, respectively. The delay time $T_{delay} = d/v \approx 3$ min, where d is the distance between end points of DNA zipping in two conditions and v is the SMC extrusion rate determined in Supplementary Fig. 4b.

(b) Experimental Hi-C maps for the control (no collision) and the one-sided head-to-tail collision at indicated time points shown in Fig. 5a and Fig. 5b, respectively. The strain contained a single *parS* site at -59°. DNA zipping progression in the control and the collision are indicated with black dashed lines and yellow dashed lines, respectively. SMC extrusion rates, v1 and v2, were determined by dividing terminus-directed and origin-directed traveling distance by traveling time (5 min), respectively. d1 and d2 are the delaying distances towards the terminus and the origin, respectively. The averaged delay time $T_{delay} = (d1/v1+d2/v2)/2 \approx 2$ min.

(c) SMC enrichment decreased beyond the collision site in the one-sided head-to-tail collision. Left and middle panels: experimental Hi-C maps for the control (no collision) and the one-sided head-to-tail collision shown in Fig. 5a and Fig. 5b, respectively. Right panel: a comparison of SMC ChIP enrichment profiles from samples shown on the left. ChIP enrichments (ChIP/input) were plotted in 10-kb bins. The positions of stalled replication forks are indicated by red dashed lines.

Supplementary Figure 6

a

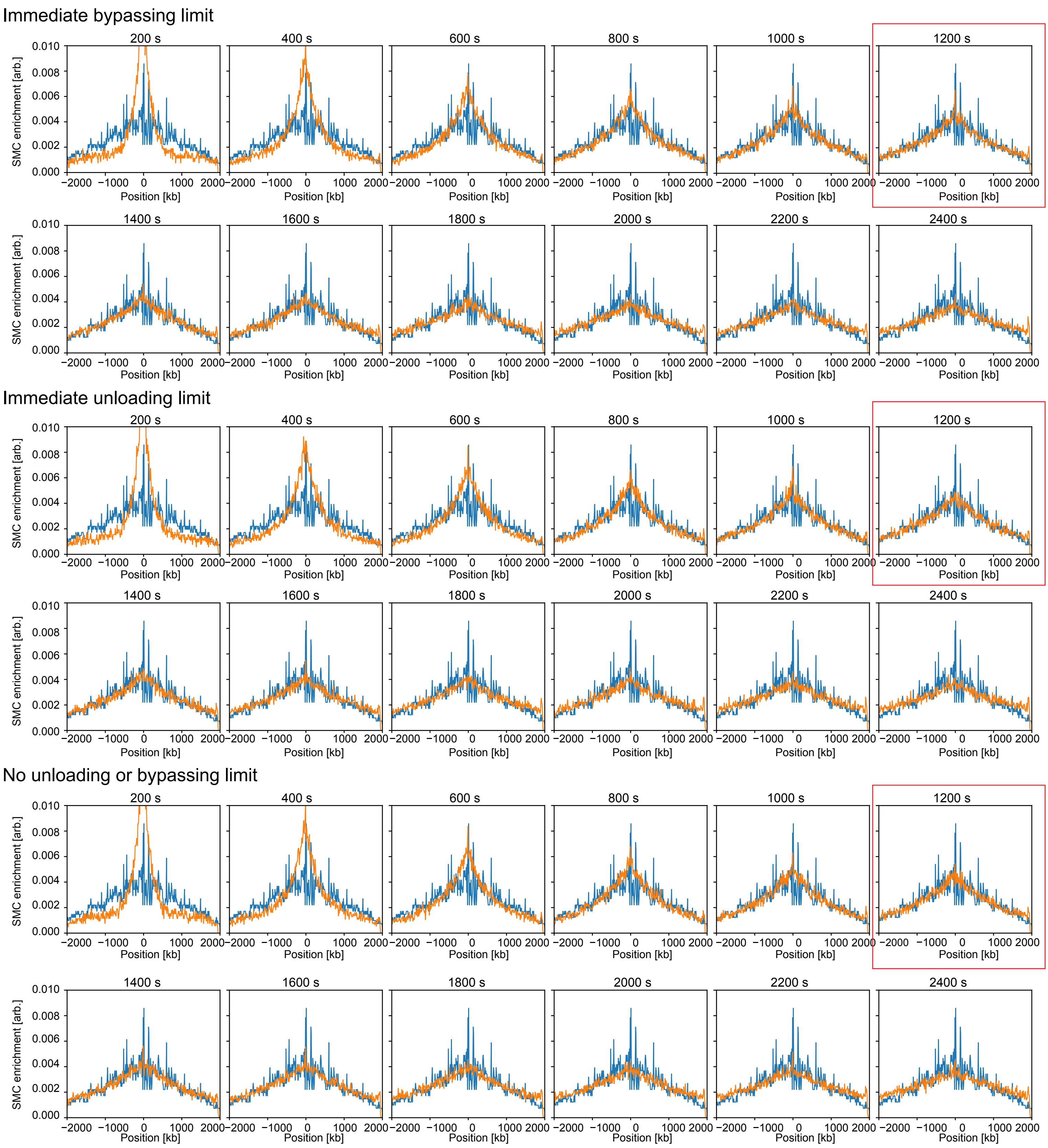

b

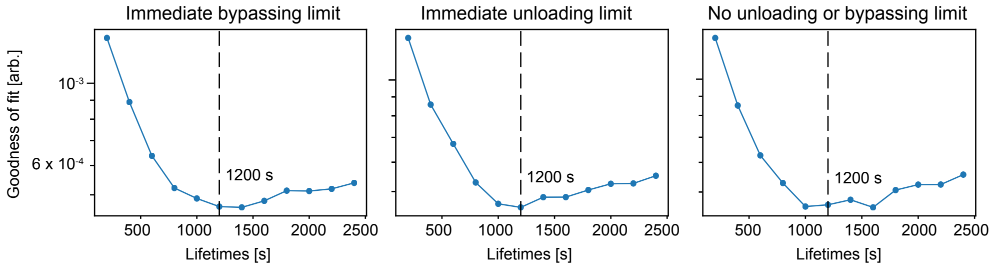

Supplementary Fig. 6: Calibration of SMC spontaneous disassociation rate at 42°C. Related to Fig. 6.

(a) SMC occupancy (orange curves) was simulated and compared with experimental ChIP-seq result (blue curves) that was obtained using G1-arrested cells growing at 42°C. This strain contained a single *parS* site at -1° and IPTG-inducible *sirA* (BWX4504), which inhibited replication initiation after 1 h of 1 mM IPTG addition. Since a small portion of cells had pre-existing replisomes as indicated in MFA results, we considered three scenarios of SMC-replisome interaction upon the encounter: 1) SMC bypasses the replisome; 2) SMC unloads from the chromosome; 3) SMC is blocked by the replisome. Simulated SMC occupancy was obtained by varying the SMC spontaneous dissociation rate from 1/200 s^{-1} to 1/1200 s^{-1}. See details in the Supplementary Methods under "calibration of spontaneous dissociation rate of SMC complexes".

(b) Goodness-of-fit analysis to compare the deviations between simulated SMC enrichments and the experimental ChIP data shown in (**a**). The best fit is the one with the minimum score. Combined with visual inspections of SMC enrichment profiles, we chose 1/1200 s^{-1} as the spontaneous dissociation rate in all simulations.

Supplementary Figure 7

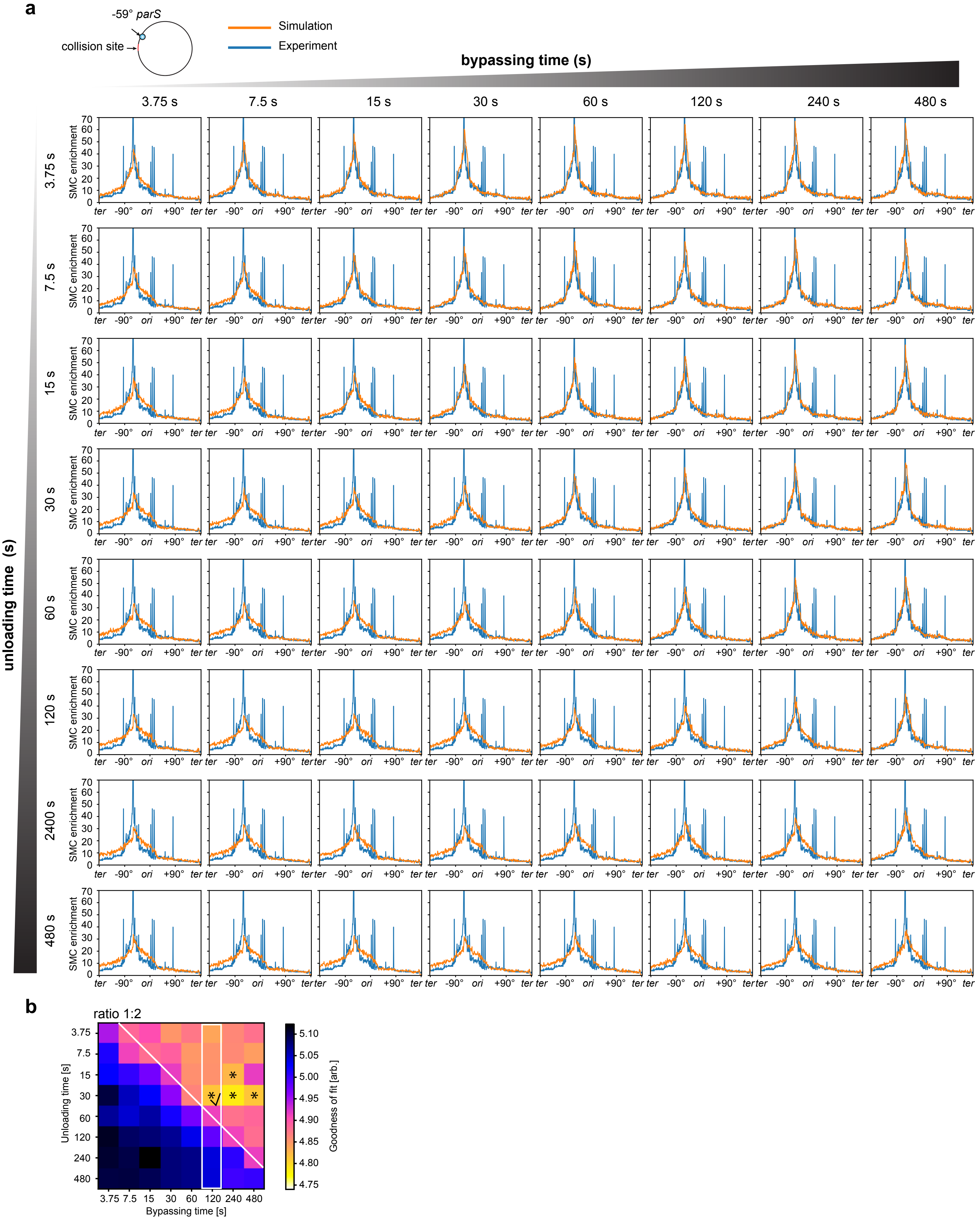

Supplementary Fig. 7: Sweeping of bypassing rates and unloading rates in the one-sided head-to-tail collision. Related to Fig. 6a-e.
(a) SMC occupancy (orange curves) was simulated by varying bypassing time and unloading time, and compared with the experimental ChIP-seq result (Fig. 5b, +IPTG 25 min time point; Fig. 6a) (blue curves). See details in Supplementary Methods under "parameter sweeps for interaction rules between SMC and the stalled replisome".
(b) A goodness-of-fit heatmap generated from simulations in (**a**). The white diagonal line indicates the 1:2 ratio of unloading time to bypassing time. The four combinations that produced the best fit are highlighted with stars. Since the experimental Hi-C analysis estimated the bypassing time to be about two minutes for this one-sided head-to-tail collision (white rectangle), the best combination in the range is unloading at 30 s and bypassing at 120 s (black check mark).

Supplementary Figure 8

a

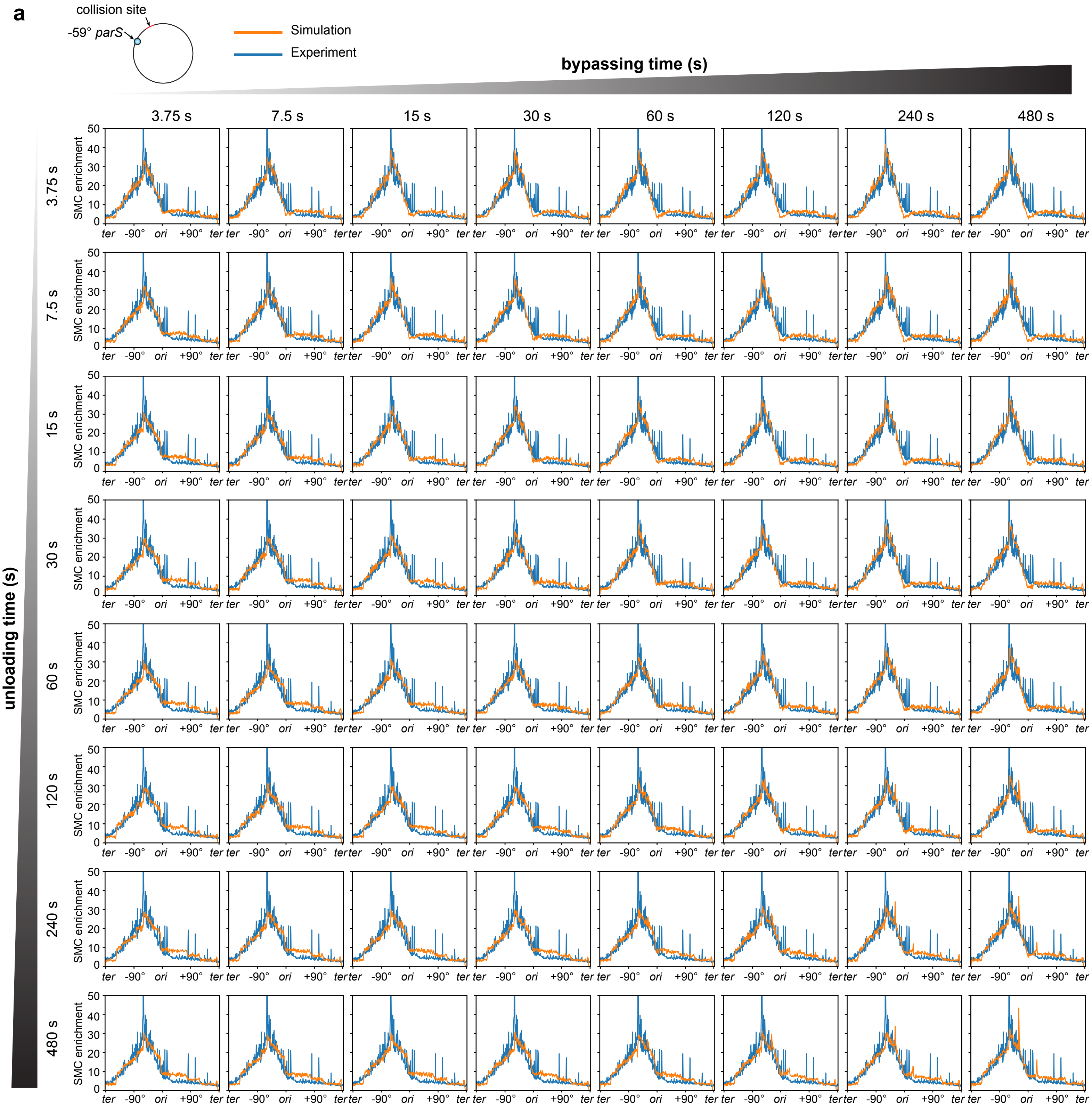

b

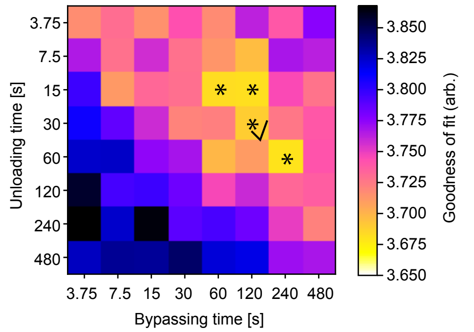

Supplementary Fig. 8: Sweeping of bypassing rates and unloading rates in the head-on collision. Related to Fig. 6f-j.

(a) SMC occupancy (orange curves) was simulated by varying bypassing time and unloading time, and compared with the experimental ChIP-seq result (Fig. 5c, +IPTG 25 min time point; Fig. 6f) (blue curves). See details in Supplementary Methods under “parameter sweeps for interaction rules between SMC and the stalled replisome”.

(b) A goodness-of-fit heatmap generated from simulations in (**a**). The four combinations that produced the best fit are highlighted with stars. The same combination used for the head-to-tail collision in Supplementary Fig. 7 (unloading = 30 s and bypassing = 120 s) is indicated by a black check mark.

Supplementary Figure 9

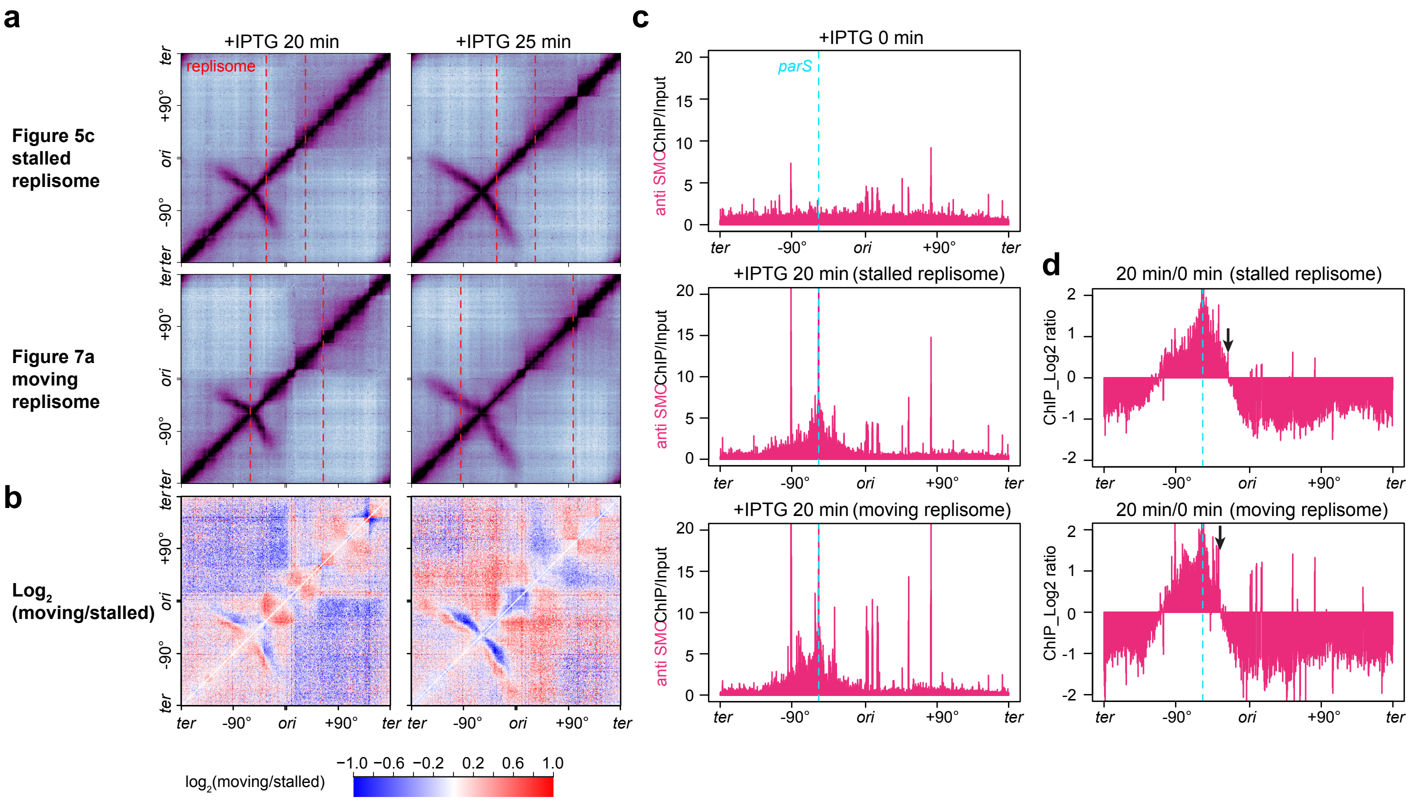

Supplementary Fig. 9: Moving replisomes have a stronger effect on SMC translocation than stalled replisomes. Related to Figs. 5c and 7a.

(a) Hi-C maps obtained from SMC collisions with stalled replisomes (top, Fig. 5c, time points +IPTG 20 min and 25 min) and from SMC collisions with moving replisomes (bottom, Fig. 7a, time points + IPTG 20 min and 25 min). Red dashed lines indicate the position of replisomes determined by MFA plots.

(b) Log_2 ratio of Hi-C matrices between "moving replisome" and "stalled replisome" shown in (**a**). Red pixels designate increased interactions while blue pixels indicate decreased interactions.

(c) anti-SMC ChIP enrichment before IPTG induction (top), or after 20-min IPTG induction in Fig. 5c which had stalled replisomes (middle), or 20-min IPTG induction in Fig. 7a which had moving replisomes (bottom). Cyan dashed lines indicate the position of the *parS* site.

(d) The ratio of ChIP enrichment at 20 min relative to 0 min were plotted in log_2 scale in 5-kb bins to track SMC translocation after IPTG induction. The top plot used cells containing stalled replisomes and the bottom plot used cells containing moving replisomes. Black arrows indicate the endpoints of SMC enrichment toward the replication origin.

Supplementary Figure 10

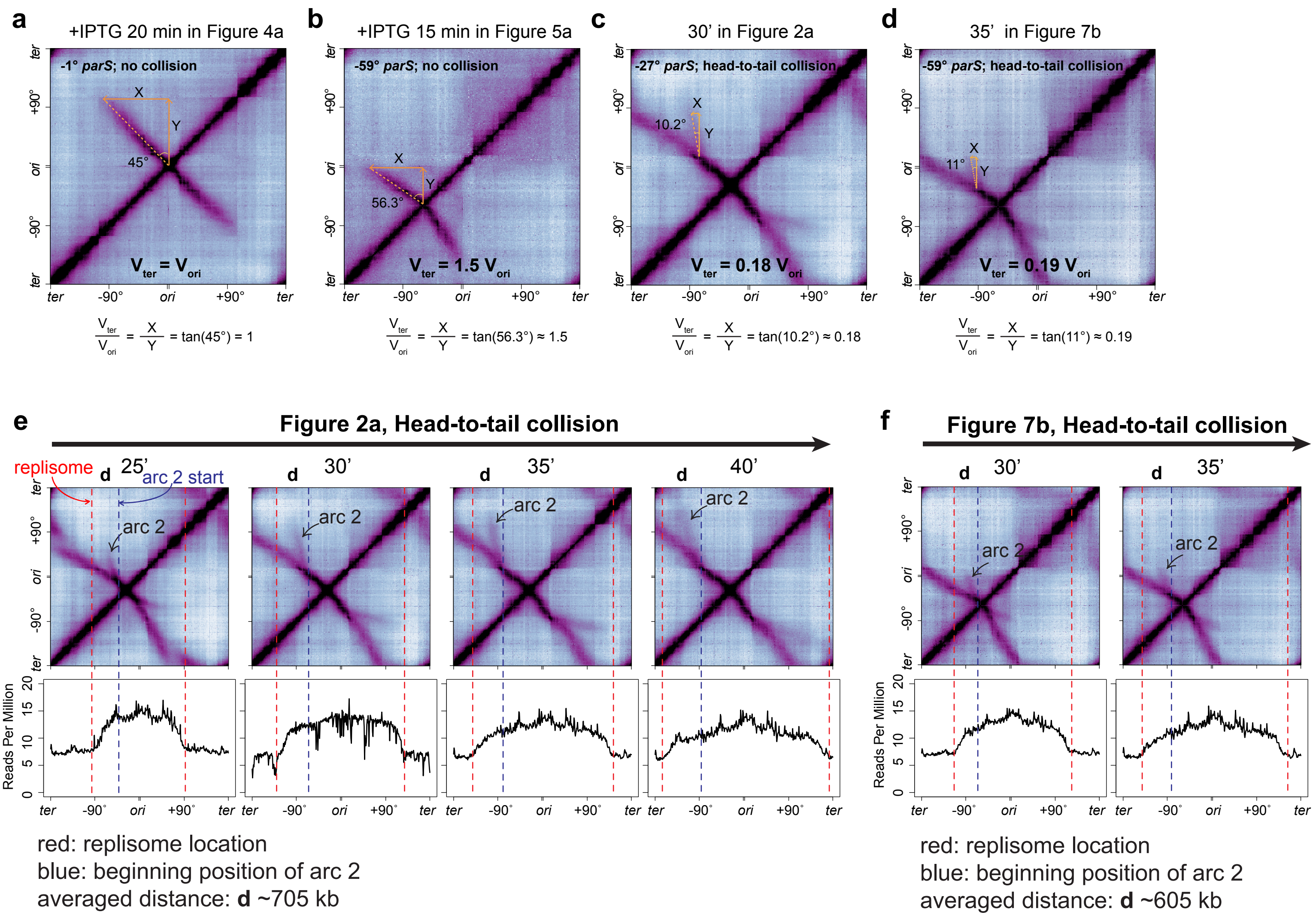

Supplementary Fig. 10: SMC was further slowed down by moving replisomes in the head-to-tail collision. Related to Fig. 7.
(a-d) The angle of DNA zipping on the Hi-C map represents the relative speed of the two independent SMC motors. X and Y indicate the translocation distance on the x-axis (toward the terminus) and y-axis (toward the origin), respectively. Shown are experimental Hi-C maps of strains containing a single *parS* site at -1° (no collision in Fig. 4a), -59° (no collision in Fig. 5a), -59° (the head-to-tail collision in Fig. 7b) and -27° (the head-to-tail collision in Fig. 2a) at indicated time points.
(e-f) Distances between the leftward moving replisome (red dashed lines) and the starting point of arc 2 (blue dashed lines) in the head-to-tail collision in Fig. 2a and Fig. 7b.

Supplementary Figure 11

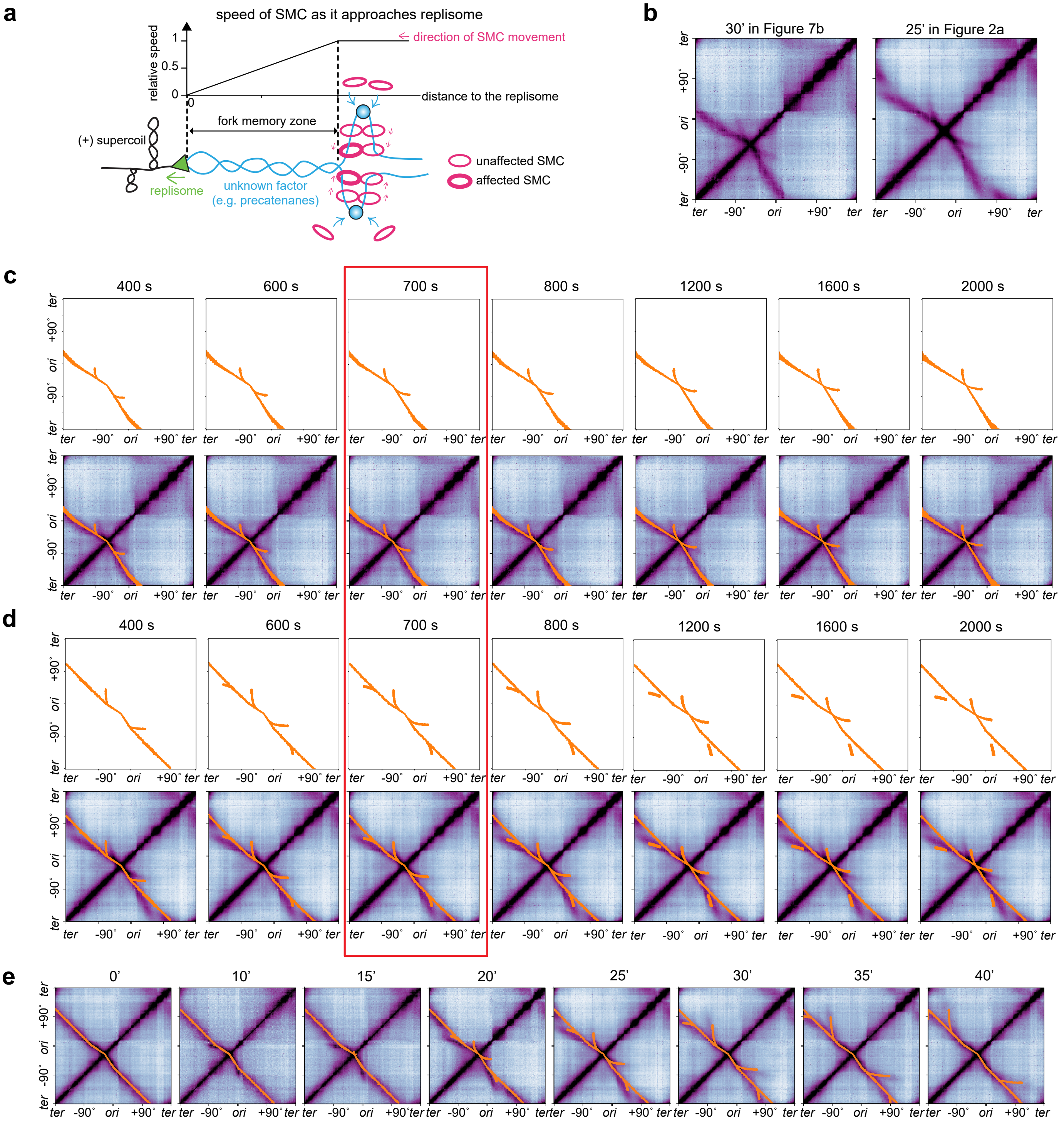

Supplementary Fig. 11: Simulations to generate arc 2 on Hi-C maps. Related to Figs. 2 and 7.
(a) Schematics depicting the setup of the model, in which SMC is progressively slowed down towards the moving replisome in a head-to-tail collision. The factor that causes such a slowdown is unknown, but an example is DNA precatenation behind the replisome. The bottom panel illustrates a moving replisome (green triangle) generating positive supercoiling ahead and precatenanes behind. In our model, SMCs loaded at *parS* behind the replisome is initially unaffected and travel at 1x relative speed. As SMCs enter the fork memory zone (e.g. where precatenanes are), SMC's speed decreases linearly and drops to zero when SMCs reach the replisome. The "fork memory time" constant describes the time SMC takes to traverse the fork memory zone. Our simulations seek to determine the fork memory time that best reproduces arc 2 in our experiments.
(b) Experimental data for head-to-tail SMC collisions with moving replisomes shown in Fig. 7b (30') and Fig. 2a (25'). Our parameter sweeps of the fork memory time in (**c**) and (**d**) seeks to reproduce the arc 2 in these two Hi-C maps.
(c) Sweeping of the fork memory time to reproduce arc 2 in Fig. 7b (30'), the left panel in (**b**). Top: at 30' after replication, with the indicated fork memory times, the final position of each SMC complex was simulated and plotted as an orange dot. A wide range of fork memory times (100 s to 4,000 s) were simulated but only a subset of those are shown. 4,000 SMC complexes are plotted. Because of such a high number of sampled SMC complexes, the dots are not individually resolvable but form lines. See Supplementary Methods for details. Bottom: overlay of dot plot (simulated positions of SMCs) on top of experimental Hi-C data.
(d) Sweeping of the fork memory time to reproduce arc 2 in Fig. 2a (25'), the right panel in (**b**) in the same way as in (**c**)**.** For both (**c**) and (**d**), simulations using a fork memory time of 600-800 s show good agreements with the experimental data in terms of the position and curvature of arc 2. The red box highlights the best fit (700 s).
(e) A fork memory time of 700 s reproduced the position and curvature of arc 2 on Hi-C maps for all time points in Fig. 2a.

Supplementary Table 1. Bacterial strains, plasmids and oligonucleotides used in this study.

**Strains used in the study**			
**Strain**	**Genotype**	**Reference**	**Figure**
PY79	Wild-type	[2]	1b
BWX3212	*parSΔ9 no a.b.*	[3]	1b
BWX3381	*parSΔ9 no a.b., -117˚ parS (loxP-kan-loxP)*	[1]	1b
BWX5297	*parSΔ9 no a.b., -59˚ parS no a.b, ΔparB::spec, yvbJ::Pspank parB (ΔparS) (cat), dnaB134 (ts) zhb-83::Tn917 (erm)*	this study	1c, 5, 6af, 7a, S1cef, S2, S4bc, S5bc, S7, S8, S9, S10b
BWX5230	*parSΔ9 no a.b., -27˚ parS no a.b., dnaB134 (ts) zhb-83::Tn917 (erm)*	this study	2a, S3, S10ce, S11bde
BWX2533	*yycR::tetO48 (cat), ycgO::PftsW tetR-cfp (phleo), dnaX-yfp (spec), dnaB134 (ts) zhb-83::Tn917 (erm)*	[3]	3
BWX4310	*parSΔ9 no a.b., ΔparB (remains wt parS at -1˚)::spec, yvbJ::Pspank parB (ΔparS) (cat), dnaB134 (ts) zhb-83::Tn917 (erm)*	this study	4, S1d, S4, S5a, S10a
BWX4504	*parS at -1˚, yhdG::Phyperspank-(optRBS)-sirA (phleo)*	this study	S6
BWX5529	*parSΔ9 no a.b., -59˚ parS no a.b, dnaB134 (ts) zhb-83::Tn917 (erm)*	this study	7bc, S10df, S11bc
**Strains used for strain building**			
**Strain**	**Genotype**	**Reference**	**use**
KPL69	*trpC2, pheA1, dnaB134 (ts) zhb-83::Tn917 (erm)*	[4]	All strains containing *dnaB(ts)*
BWX2423	*ΔparB (ΔparS)::loxP-spec-loxP*	[5]	BWX5297
BWX3268	*parSΔ9 no a.b., -27˚ parS no a.b.*	[1]	BWX5230
BWX3370	*parSΔ9 no a.b., -1˚ parS no a.b.*	[1]	BWX4504
BWX3377	*parSΔ9 no a.b., -59˚ parS (loxP-kan-loxP)*	[1]	BWX5297
BWX4070	*parSΔ9 no a.b., ΔparB (remains wt parS at -1˚)::spec, yvbJ::Pspank parB (ΔparS) (cat)*	[6]	BWX4310
BWX4426	*parSΔ9 no a.b., -59˚ parS no a.b*	this study	BWX5297
BWX4464	*parSΔ9 no a.b., -59˚ parS no a.b, ΔparB::spec*	this study	BWX5297
BWX4467	*parSΔ9 no a.b., -59˚ parS no a.b, ΔparB::spec, yvbJ::Pspank parB (ΔparS) (cat)*	this study	BWX5297
**Plasmids used in the study**			
**Plasmid**	**Description**	**Reference**	**use**
pDR244	*cre*-expressing plasmid *(spec)*	[7]	BWX5297
pJW005	*yhdG::Phyperspank-(optRBS)-sirA (phleo)*	[8]	BWX4504
pWX722	*yvbJ::Pspank (optRBS) parB ΔparS (cat)*	[6]	BWX4310, BWX5297
**Oligonucleotides used in the study**			
**Oligos**	**Sequence**	**Reference**	**Use**
oWX507	cgtgcttgaattttcaattatttccc	this study	BWX5297
oWX508	acccgttgcaaaggctcactgggcgc	this study	BWX4310
oWX888	agaggtaaacgtaatgctcgcaggcc	this study	BWX5297

Supplementary Methods

Strain construction

-59° *parS*, Δ*parB*, *Pspank parB without parS, dnaB(ts)* (BWX5297)

This strain was constructed in four steps: 1) The *loxP-kan-loxP* cassette in BWX3377 [1] was removed using a *cre*-expressing plasmid pDR244 [7], resulting in BWX4426; 2) A PCR product of Δ*parB* clean deletion was amplified using oWX507 and oWX888 from strain BWX2423 [5] and transformed into strain BWX4426, resulting in BWX4464; 3) pWX722 (*Pspank parB without parS*) [6] was transformed into BWX4464, resulting in BWX4467; 4) The genomic DNA of *dnaB134 (ts) zhb-83::Tn917* [4] was transformed into BWX4467.

-59° *parS, dnaB(ts)* (BWX5529)

This strain was constructed by transforming the genomic DNA of the *dnaB134 (ts) zhb-83::Tn917* strain [4] into BWX4426 described above.

-27° *parS, dnaB(ts)* (BWX5230)

This strain was constructed by transforming the genomic DNA of the *dnaB134 (ts) zhb-83::Tn917* strain [4] into BWX3268 (*parS*Δ*9 no a.b., -27˚ parS no a.b.*) [1].

-1° *parS*, Δ*parB*, *Pspank parB without parS, dnaB(ts)* (BWX4310)

This strain was constructed by transforming the genomic DNA of the *dnaB134 (ts) zhb-83::Tn917* strain [4] into BWX4070 [6].

-1° *parS*, *Phyperspank sirA* (BWX4504)

This strain was constructed by transforming pJW005 [8] into BWX3370 [1].

Calibration of replisome dynamics model

We first simulated replisome dynamics in a population of synchronously growing cells. Our objective was to reproduce the shape of the marker-frequency analysis (MFA) as determined by whole-genome sequencing (WGS) to define the replisome distribution on the genome using the fewest parameters possible.

We used the MFA data from the *dnaB(ts)* strain (BWX5297) in Fig. 1c. We performed simulations to vary several parameters on replisome behaviors: 1) the loading rate of DNA polymerase (DNAP) to the origin (*DNAP_load_time*); 2) the speed of replication forks at 30°C (*low_temp_DNAP_rate*); 3) the speed of replication forks at 42°C (*high_temp_DNAP_rate*); 4) the time for spontaneous DNAP stalling (*DNAP_stall_time*); 5) the fraction of cells containing replisomes before initiation (*fraction_pre-existing DNAP*). See below for detailed explanations.

In the experiment in Fig. 1c, cells were first synchronized at the G-1 stage by being grown at 42°C for 45 min (T = 0 min). The overall "flat" shape of the curve in the MFA profile at this time point (Fig. 1c, 0') indicates that most of the cells in the synchronous population contained a single chromosome. However, the slight "hat shape" (i.e. ^) of the curve (Supplementary Fig. 1a, slope 1) indicates that some cells had partially replicated chromosome prior to replication initiation, despite growing at 42°C. We refer to the replisomes in this subpopulation as pre-existing replisomes, heuristically modeled its fraction by the *fraction_pre_existing_DNAP* parameter, and distributed these replisomes using a wide quadratic distribution centered with its maximum at the origin.

For experimental data upon replication initiation, we found that the shapes of the MFA distribution were best matched by including a parameter, *DNAP_stall_time*, to account for spontaneous DNAP stalling. This parameter modeled the probability that a replisome prematurely stops, which could be caused by factors such as DNA damage or other temporary roadblocks. The spontaneous stalling time was modeled using an exponential distribution with the characteristic stalling rate governed by 1/*DNAP_stall_time*.

We combined all these elements into a function to compute the probability distribution of DNAP positions

along the chromosome at given time points. Briefly, we uniformly sampled the loading times for each DNAP from an exponential distribution with average time *DNAP_load_time* to pre-determine the loading time of each replisome (DNAP). Additionally, we sampled *DNAP_stall_time* from an exponential distribution with average time to determine the spontaneous stalling time of each replisome (DNAP). We then paired up all possible combinations of sampled DNAP loading times and stalling times to generate a minimum number of sampled (simulated) replisomes.

Upon replication initiation, for simplicity, we assumed that replisomes progressed symmetrically to the left and right of the origin. Each replisome traveled away from the origin using the rate *low_temp_DNAP_rate* for the first 15 min when the cells were grown at 30˚C, followed by *high_temp_DNAP_rate* when growing 42°C. The distance traveled by each replisome on each replication arm was computed by (*low_temp_DNAP_rate* ∗ 10 min or 15 min and *high_temp_DNAP_rate* ∗ time for time T-15 min). A fraction of pre-existing chromosomes was additionally distributed as described above.

This model for the distribution of replisome positions was then normalized and compared to experimental data. To fit the model parameters, we employed a least-squares optimization approach to collectively minimize the difference between the distributions of simulated replisome position and the experimental MFA data at the time points 0 min, 10 min, 20 min, 30 min, 40 min, 50 min after replication initiation. Both simulation and experimental MFA data were binned into 10 kb segments for a total of 404 bins for the whole genome. We normalized both the experimental data and the simulated copy number by the sum of total counts, then multiplied the values by 4200. This was calculated by: 42 ∗ 1e6/1e4 = 4200. The factor of 42 comes from the fact that the WGS read length is 42 bases long; the 1e6 is for reporting values in reads per million; finally, we divided the total by 1e4 since the data was median filtered with a window size of 10 kb. For the least-squares optimization, we used scipy1.4.1 (scipy.optimize.minimize). By sweeping the parameters above, we identified a set of best-fit values that reproduced the experimental MFA data in Fig. 1c (also see Supplementary Fig. 1b and c).

Parameters	Values	Units	Fit/Fixed
DNAP_load_time_constant	5	1/min	fit
low_temp_DNAP_rate	22	kb/min	fit
high_temp_DNAP_rate	66.0	kb/min	fit
DNAP_stall_time_constant	44	1/min	fit
fraction_pre_existing_DNAP	0.18	N/A	fit
num_chromosomes_for_fit	11800	N/A	fixed
bin_size_for_fit	10	kb	fixed
chromosome_length_in_bins	404	kb	fixed
time_for_DNAP_loading	15	min	fixed
time_at_low_temperature	15	min	fixed

From these fit values, we estimated that within a 15-minute time window at 30˚C, approximately 5% of the cells do not initiate DNA replication (i.e., exp(-15min/DNAP_load_time_constant) = exp(-15min/5min) ~ 0.05 = 5%). Therefore, ~95% of cells were estimated to have initiated replication.

Using the same fitting procedure and same parameters, our simulation reproduced the experimental data in other experiments used in this study (Figs. 4, 5 and Supplementary Fig. 1d-f). Although it is mechanistically not precise, our heuristic model could nevertheless provide insights into the nature of the replisome dynamics. Our simulations provided the following estimates (mean ± standard error on mean):

DNAP load time constant = 4.7 ± 0.4 min
Low temperature DNAP rate = 27 ± 3 min
High temperature rate = 57 ± 4 min
DNAP_stall_time_constant = 64 ± 20 min
Fraction pre-pre-existing DNAP = 20 ± 2 %

Framework for simulations of SMC extrusion on replicated chromosomes

To assess the possible rules of the interaction between loop-extruding SMC complexes and replisomes, we extended our previous framework for simulations of SMC-SMC interactions [9] (https://github.com/hbbrandao/bacterialSMCtrajectories) by including replicated chromosomes and SMC-replisome interactions.

Each simulated chromosome was segmented into 4040 bins of 1 kb each to resemble the total length ~4033 kb in our bacterial strains and to best match the number of 404 bins of 10 kb used for Hi-C and other analyses. In addition to the 4040 bins for the main (unreplicated) chromosome present in all simulations, we also added a number of lattice sites for the segments of chromosome that have been replicated. As such, each simulation could contain up to 4040 × 2 = 8080 chromosomal lattice sites to allow for up to two whole replicated chromosomes per cell. At the beginning of each simulation, replisome positions were assigned based on *fraction_pre_existing_DNAP* (see above).

For all simulations, we allowed the number of SMCs on the chromosome to fluctuate based on their loading/unloading dynamics, which was done by including a "cytosolic lattice site" for SMC to reside in addition to the aforementioned chromosomal lattice sites. We used a constant number of 40 SMC loop-extruding molecules per cell as determined by previous experiments and simulations [9]. At the start of each simulation, ~75% of SMCs (30 loop extruders) started off at the "cytosolic" locus to account for the fact that SMCs have not yet been induced to load on the DNA. ~25% (10 loop extruders) were "pre-loaded" throughout the chromosome to mimic the non-specific loading of SMCs in the absence of *parS* sites or ParB protein [9, 10].

For simulating SMC dynamics, we created a Python program defining a smcTranslocator class as the engine for simulating the motion of SMC complexes on the replicating chromosome. This class encapsulated the dynamic behaviors of SMCs, including their loading onto the DNA, their translocation along the chromosome, and their unloading. Broadly, the simulation of SMC dynamics can be broken down into three main parts ordered by when they occur in each simulation time step: **death**, **birth** and **step**:

death: This function governs the unloading or dissociation of SMCs from the chromosome. It includes both the basal unloading rate which represents the intrinsic tendency of SMCs to dissociate from the DNA, and the possibility of unloading triggered by interactions with other SMC complexes or interactions with the replisome. Death probabilities were pre-set at the start of each simulation and were calibrated for each simulation time-step to specific average lifetimes for the SMCs at each type of lattice site. For the cytosolic lattice site, the average lifetime was calibrated to be 180 s. For the chromosomal lattice sites including the "*parS* lattice site" and "regular lattice sites", the average lifetime of the SMC was set to 1200 s as determined from experiments (see the SMC lifetime calibration section below for details). For "replication fork lattice sites", the average lifetime (or probability of unloading) was left as a free parameter for each simulation (i.e. we performed parameter sweeps over this value with values ranging from 1 s [the case of immediate unloading] to 10,000,000 s [the case of no unloading]). Finally, we also defined a set of 100 terminus lattice sites (i.e. lattice sites 1950 to 2050 at the replication terminus region) to mimic the unloading of SMCs [11]. An SMC at a *ter* site had a probability of unloading of 10% per simulation time step.

birth: This function orchestrates the transfer of dissociated SMCs onto one of the three possible simulated lattice sites described above (i.e. two for the chromosome, or one for the cytoplasm). The loading probabilities are pre-set at the start of each simulation by defining lattice sites as one of: "cytosolic" (loading strength 16,000), "*parS*" (loading strength 4000), "replication fork" (loading strength 1) or "regular" (loading strength 1). As an example, for a fully replicated chromosome has 8080 chromosomal lattice sites, including two *parS* sites (one for each daughter DNA molecules), and 2 × 4039 regular chromosomal lattice sites, separate from the lattice site for cytoplasmic SMCs. In this example, an SMC undergoing the "birth" (or loading) operation would be randomly selected to bind to the cytoplasmic lattice site with probability 16000/(16000 + 2 × 4000 + 4039 × 1 × 2) = 49.9%, to a *parS* site with probability (2 × 4000)/(16000 + 2 × 4000 +

4039 × 1 × 2) = 24.9%, and to any other regular site with probability 1/(16000 + 2 × 4000 + 4039 × 1 × 2) = 0.003% .

step: This function simulates the translocation of SMCs along the chromosome. For each simulation step, it considers various factors influencing SMC movement, such as directionality, translocation rate and stalling probabilities. The step function uses periodic boundary conditions to model SMC movement on a circular chromosome. When an SMC moves past one end, it is repositioned to the other, creating a continuous track for translocation, reflecting the circular nature of bacterial chromosomes. In our simulations, each SMC loop extruder consists of two "motor subunits" that can move independently from one another [12], and interacts independently with other SMCs and the replisome. By default, the rule of engagement of an SMC with a replisome is "pausing" (also known as blocking or doing nothing). If an SMC subunit translocating on unreplicated DNA in *ter*-to-*ori* orientation needs to bypass (step over) a lattice site containing the replication fork (head-on collisions between SMC and replisome), the "step" function randomly selects one of the two newly replicated daughter DNA strands to move to with probability of 50% for either one. For the reverse cases where an SMC subunit moving on newly replicated DNA strands in an *ori-to-ter* direction encounters a replication fork site ahead (head-to-tail collision), we assumed SMCs move from the daughter DNA to the unreplicated mother DNA (i.e. transitions from daughter DNA to daughter DNA are not allowed). For the rules of engagement of SMCs with other SMCs, we fixed the rates of pausing (probability of 0.9275 per simulation step), bypassing (probability of 0.07 per simulation step) and facilitated dissociation (probability of 0.0025 per simulation step) as previously described [9]. Lastly, to best mimic the asymmetric translocation dynamics of the SMC complexes, we specified a genomic bias for the translocation: SMCs translocating from lattice site 90 (i.e. position 90 kb from *ori*) towards the terminus (lattice site 2000) move at the "full speed", and from lattice site 2000 to 90 at 33% of the full speed to reproduce the correct "tilt" in the Hi-C secondary diagonal [12]. The full speed of SMC used in the simulations was 1.07 kb/s at 42˚C, close to the value of 1.18 kb/s as measured by experimental Hi-C (Supplementary Fig. 4b), and was 0.35 kb/s at 30˚C as inferred by fitting a time-course of SMC ChIP-seq data (Supplementary Fig. 2b). All simulation time step units were calibrated to an equivalent of one second of real experimental time.

We note that all of the above simulations of SMC extrusion dynamics are one dimensional in nature.

Calibration of spontaneous dissociation rate of SMC complexes

We constrained the spontaneous dissociation rate of SMCs at 42°C using SMC ChIP-seq (Supplementary Fig. 6a). We used a strain (BWX4504) containing a single *parS* site at -1° and IPTG-inducible *sirA* which arrested cells in G1 after one-hour treatment with 1 mM IPTG at 42°. In this dataset, most of the chromosomes were unreplicated, but the MFA analysis suggested that a small fraction of chromosomes in the cell population had pre-existing replisomes in a similar way as seen in MFA data of the *dnaB(ts)* strain described above (see the section *calibration of replisome dynamics model)* (Supplementary Fig. 1a, slope 1). As such, we took several steps to ensure that our estimate of the spontaneous SMC dissociation rate was not strongly biased by this fraction of pre-existing replisomes. We took the following key steps to determine the spontaneous SMC dissociation rate:

1. **Data preparation:** Loading and binning experimental ChIP-seq and MFA data.
2. **Replisome simulation:** Because the cells were arrested in G1, we set the DNAP loading time to 100,000 min, which was effectively not allowing any replisomes to load on the DNA. Using the fitting procedure we described in "Calibration of replisome dynamics model", we identified that 5% of chromosomes had pre-existing replisomes. We considered their effects on SMC dynamics (see below).
3. **SMC translocation simulations:** We implemented a function (smcTranslocator) to simulate SMC movement on the chromosome, incorporating factors like bypassing, unloading, and pausing at the replication fork positions. We performed four simulations to account for the 5% of pre-existing replisomes. We first simulated the three extreme interaction scenarios between SMC and the replisome: 1) "blocking" where SMC translocation is blocked by the replisome and SMC remains at the collision site; 2) "unloading" where SMC immediately dissociates from the

chromosome when encountering the replisome; 3) “bypassing” where SMC immediately bypasses the replisome. We reasoned that if all the fit values for these extreme scenarios gave similar “best-fits”, then the residual 5% of pre-existing replisomes would have an overall small effect on the estimated SMC dissociation rate. As a final sanity check, we ran a fourth set of simulations assuming there was no residual (i.e. partially replicated) chromosome.

4. **Parameter sweep:** We systematically varied the SMC lifetime (i.e. 1/ (spontaneous dissociation rate)) to generate a series of simulated SMC enrichment profiles. We swept lifetime values from 200 s to 2,400 s at intervals of 200 s. The resulting simulated SMC profiles were then compared directly to the experimental ChIP-seq data (Supplementary Fig. 6a). We inspected the results both visually and by computing a goodness of fit metric.
5. **Goodness-of-fit analysis:** We compared simulated SMC enrichment profiles with experimental data to identify the SMC dissociation rate. The goodness-of-fit metrics we employed was the mean absolute deviation between the simulated SMC profiles and the experimental ChIP-seq data (Supplementary Fig. 6b). By considering the goodness-of-fit curves for all three extreme-value scenarios of SMC-replisome interaction rules, we found that the overall best fit value for the average SMC lifetime was ~1,200 s.

Generation of SMC occupancy profiles from simulations
Each SMC loop extruder has two motors (the “left” extrusion motor and the “right” extrusion motor). Thus, at each simulation time step, the position of one SMC loop extruder has a “pair” of positions corresponding to the two motors. For each lattice site, we counted the number of instances when a SMC motor was on it. The counts were summed for all SMCs on all chromosomes in a simulation (typically at least 1,025 chromosomes) to produce the plotted SMC occupancy profiles (i.e. simulated ChIP). As done for experimental ChIP-seq data, we discretized the simulated genome into 404 bins (each consisting of 10 lattice sites) and plotted the median SMC occupancy count value within each 10 kb bin. Lastly, we normalized the entire distribution by the area under the curve (i.e. the sum of all 404 median bin counts) and multiplied the result by 4,200 to report the simulated SMC occupancy profile in reads per million. In this way, we can directly compare the simulated SMC enrichments to the experimental ChIP enrichments.

Generation of Hi-C maps from simulations
For generating Hi-C maps from simulations, we used the semi-analytical methods described in Brandao et al, 2021 [9] which builds on the work from Banigan et al, 2020 [13]. Briefly, the simulated Hi-C maps come from calculations of the contact probabilities between different parts of the chromosome (i.e. the chromosomal lattice sites). Since these contacts are mediated by the two motors of SMCs, the method used positions of the loop extruding SMC complexes on the genome to compute an undirected graph to obtain the shortest path between any two randomly sampled chromosomal lattice sites. We used Scipy1.4.1 (scipy.sparse.csgraph.shortest_path) for the shortest path calculation.

To create the simulated Hi-C maps shown in Fig. 6 we sampled at least 5,152 chromosomes for each map, with 1,000,000 randomly sampled unique contact pairs for each chromosome. Sections of the genome with replicated chromosomes were averaged. Contacts were binned to 10 kb resolution, resulting in a single Hi-C contact map of 404 bins, which was comparable to the experiments. We note that it was not possible to directly compare the Hi-C contact values in the simulated map with those in the experimental data because of the shortest-path approximation [9], Gaussian contact model approximation [13], and the lack of simulated “fine structures” such as plectonemes [14]. To closely match the experimental contact probability decay and to show the Hi-C maps on comparable color scales, we adjusted the scaling exponent in the Hi-C simulations. This adjustment aids visual comparison but does not alter our results or conclusions.

Filtering of ChIP-seq and MFA data for goodness-of-fit calculations and visual comparisons
All ChIP-seq and MFA data were mapped to 1 bp resolution. We then applied a 10 kb moving “window” and measured the median value within the window. Data were discretized to 404 bins to cover the entirety of the genome similarly to the process described above for simulated SMC occupancy profiles. For direct comparisons of the simulated SMC enrichment profiles to experimental ChIP-seq data, we did not normalize the ChIP by the DNA input (i.e. MFA data) because of two reasons: 1) our simulations already account for the distribution of multiple chromosome copies; 2) since MFA data are noisy, we

reasoned that comparing the ChIP-seq data directly to the simulated profiles would result in higher confidence and less bias.

Parameter sweeps for interaction rules between SMC and the stalled replisome
We took the following steps to generate parameter sweeps:

1. **Data preparation:** We loaded and binned experimental ChIP-seq and MFA data as described above. We fit the data using one timepoint specified in the figure legend (e.g. we used +IPTG 25min in Fig. 6a, f).
2. **Replisome simulation:** To mimic the distribution of replisomes for each experimental condition, we used the best-fit values that reproduced the MFA distributions of cells in Fig. 1c (see the section *Calibration of replisome dynamics model*, also see Supplementary Fig. 1b, c), which also reproduced the MFA plots for the stalled replisomes (Supplementary Fig. 1d-f).
3. **SMC translocation simulations and parameter sweep:** We employed the smcTranslocator function to simulate SMC movement on the chromosome, incorporating factors like bypassing, unloading, and blocking (i.e. pausing) at the replication fork positions. While keeping all other parameters fixed (as described above in the section *Framework for simulations of SMC extrusion on replicated chromosomes*), we systematically varied the *SMC_bypassing_time* and the *SMC_unloading_time* values. We used all pairwise combinations of the following times [3.75, 7.5, 15, 30, 60, 120, 240, 480] in seconds. Additionally, to simulate the simple models, we used the pairs of values for [*SMC_bypassing_time; SMC_unloading_time*] of i) [10,000,000; 10,000,000] for blocking only; ii) [1; 10,000,000] for immediate bypassing only; and iii) [10,000,000; 1] for immediate unloading only. We median-filtered the resulting simulated SMC profiles as done for the experiments, and then compared the simulations and experiments directly both visually and by computing a goodness-of-fit metric (Supplementary Figs. 7 and 8).
4. **Goodness-of-fit analysis:** We compared simulated SMC profiles with experimental data to identify the parameter combination that best explains the observed SMC distribution (Supplementary Figs. 7b and 8b). The goodness-of-fit metric we employed was the mean absolute deviation between the simulated SMC profiles and the experimental ChIP-seq data.

Simulations with an active replisome
In addition to the general SMC-replisome interaction rules explored with the simulation framework above, we developed a specialized, stand-alone simulation to investigate in more detail the dynamics of a co-loaded pair of SMC motors encountering an explicitly moving replication fork. In this model, at each simulation time step, not only do the SMC motors potentially move, but the position of the replication fork also advances along the lattice sites. This model focuses on two SMC motors initialized at the same starting position on the 4040-site circular lattice, with one designated to move clockwise and the other counter-clockwise as before.

To simplify the analysis and focus specifically on the new concept of “fork memory” – the lingering effect of a replication fork’s passage on the local chromatin and subsequent SMC motor movement, this specialized simulation intentionally omitted certain complexities present in the previous framework. Specifically, it did not include detailed SMC-SMC interactions (like those leading to facilitated dissociation or mutual pausing). Furthermore, direct SMC-replisome interactions such as “pushing”, “stalling”, or “unloading” upon encounter were not explicitly modeled. Instead, the primary goal was to understand how SMC motors that are assumed to be capable of bypassing the replisome are affected by a transient alteration of their translocation speed in regions recently traversed by a replication fork.

For these simulations, movement of each SMC motor in the pair was stochastic. At each simulation step, an attempt was made to move one site, governed by a rate function dependent on the motor’s current distance from the replication fork and the direction of movement relative to the fork. Two rate functions were defined as follows:

1. **Steady-state rate function for paired motors:** In the absence of a replication fork's influence, or before the replisome reaches an SMC motor's location, the SMC movement rates (i.e., the

probability of SMC advancing to the next lattice site during a simulation time-step) were spatially heterogeneous as before.

- For clockwise movement, SMC motor rate was 1.0 for positions $0 \leq p < 2020$ and 0.66 for positions $2020 \leq p < 4040$.
- For counter-clockwise movement, SMC motor rate was 0.66 for positions $0 \leq p < 2020$ (i.e., moving from 2019 towards 0) and 1.0 for positions $2020 \leq p < 4040$ (i.e., moving from site 4039 towards 2020). These differential rates modeled intrinsic regional variations in mobility for this paired configuration.

2. **Post-replication fork rate function for paired motors:** This function models how a replication fork alters the local movement rates of the paired SMC motors. The function is a linear interpolation between the steady-state rate (*R_steadystate*) and a modified rate (*R_modified*) to give a final overall rate: R_final=*R_modified* × f + (1– f) × *R_steadystate*. The factor, f, represents the strength of the fork's lingering influence. The parameter f is 0 when the motor is at or ahead of the fork front. When the motor is behind the fork, f depends on the SMC motor's current distance from the advancing replication fork front. If the motor is close to the fork front, f is close to 1, meaning the movement rate is primarily the modified rate, *R_modified*, which was typically set to 0. If the motor is further behind the fork front, f is closer to 0, signifying that the lattice site has "forgotten" the fork's passage, and the rate is closer to the steady-state value. When the motor is behind the fork, f is calculated as: f = 1 – min((*distance_of_SMC_behind_the_fork*) / (*fork_memory_distance*), 1), where *distance_of_SMC_behind_the_fork* is the SMC motor's current distance from the replication fork. The free parameter *fork_memory_distance* was the main value we were seeking to find and optimize. While *fork_memory_distance* was implemented as a characteristic distance in kilobases in the simulation code and equation above, it could also be conceptualized as a characteristic time. This temporal interpretation arises because the distance at which an SMC motor lags behind the continuously moving replication fork is related to the time elapsed since the fork passed that motor's initial relative position. Specifically, this characteristic time is given by *fork_memory_time* = *fork_memory_distance*/*v_fork*, where *v_fork* is the speed of the replication fork (i.e., 66 kb/min). This linear model was selected for its simplicity, aiming to capture the essential dynamics of a transiently altered environment with the fewest additional free parameters, allowing a focused investigation on the impact of the *fork_memory_distance* parameter.

The logic for applying the various rates of SMC movement is as follows. The simulation first checks if the replication fork originating from site 0 (with its own *fork_start_time*, an exponentially drawn number of seconds with a mean value of 5 mins) and moving at *v_fork* = 66 kb/min has reached the SMC motor's position. If not, the steady-state rates are applied. If the fork has passed the motor's position, the modified rates are applied, interpolated with steady-state rates via the factor f described above.

A time-scaling mechanism was used to map the experimental "real time" points to "effective simulation time" points. This empirical mapping was necessary because the underlying experiments were conducted at two different temperatures (30˚C and 42˚C), which resulted in different SMC translocation speeds after the 15 min time point. The mapping is as follows:

- Real time 0 min → Effective time 0 min
- Real time 10 min → Effective time 5 min
- Real time 15 min → Effective time 8 min
- Real time 20 min → Effective time 16 min
- Real time 25 min → Effective time 21 min
- Real time 30 min → Effective time 26 min
- Real time 35 min → Effective time 31 min
- Real time 40 min → Effective time 36 min

The effective simulation time (in minutes) determines the duration (in numbers of simulation steps) for which the system evolves under observations. Parameter sweeps were conducted by varying:

- The effective time (to simulate different experimental time-points).

- *fork_memory_distance* (the parameter governing the decay distance of the fork's influence).

To generate the dot plots, we simulated a total of 4,000 SMC motor pairs per condition. A single simulation run tracked one clockwise and one counter-clockwise SMC motor, typically starting from a *parS* site (e.g., site 3,378 for the *parS* at the -59˚ position or site 3,737 for the -27˚ *parS* position). The simulation initiated assuming a uniform loading time for the SMC relative to the "experimental start time" (i.e., 0 min). The simulated SMC motor pair terminated if both motors reached positions adjacent to the terminus at position 2,020 or after a maximum number of simulation steps determined by the total simulation time, which is the effective simulation time given by the experimental start time (see section above) minus the replisome loading time. When generating trajectories for the dot plots, we accounted for the fraction of the chromosomes not initiating replication. In such cases, the rates of movement of SMCs were entirely governed by the steady-state rate function. For the large majority of simulations (i.e., with an "active replisome") we used the combination of the steady-state rate function and the post-replication rate functions as defined above. The final lattice positions of the counter-clockwise and clockwise motors from each simulation run were transformed to a zero-centered chromosomal coordinate system and were plotted as in Fig. 7c and Supplementary Fig. 11. These plots aimed to reveal how different parameters governing the SMC-replication fork interaction affect the relative and absolute progress of the two motors away from their start site.

Declaration of generative AI and AI-assisted technologies in the writing process
During the preparation of this work the authors used Gemini AI 1.0 Ultra (Google Workspace, accessed September 8-15, 2024) and Gemini AI 2.5 Pro Preview (Google Workspace, accessed May 14-20, 2025) to parse our replisome simulation codes, organize and extract the names of the main variables and functions used, and generate a draft summary of the codes and methods. After using this tool, the authors reviewed and edited the content as needed and take full responsibility for the content of the publication. All subsequent writing and editing, including the details of how the simulations were performed were written by the authors.

Supplementary References

1. Wang X, Brandao HB, Le TB, Laub MT, Rudner DZ. Bacillus subtilis SMC complexes juxtapose chromosome arms as they travel from origin to terminus. *Science* **355**, 524-527 (2017).
2. Youngman PJ, Perkins JB, Losick R. Genetic transposition and insertional mutagenesis in Bacillus subtilis with Streptococcus faecalis transposon Tn917. *Proc Natl Acad Sci U S A* **80**, 2305-2309 (1983).
3. Wang X, Le TB, Lajoie BR, Dekker J, Laub MT, Rudner DZ. Condensin promotes the juxtaposition of DNA flanking its loading site in Bacillus subtilis. *Genes Dev* **29**, 1661-1675 (2015).
4. Rokop ME, Auchtung JM, Grossman AD. Control of DNA replication initiation by recruitment of an essential initiation protein to the membrane of Bacillus subtilis. *Mol Microbiol* **52**, 1757-1767 (2004).
5. Graham TG*, et al.* ParB spreading requires DNA bridging. *Genes Dev* **28**, 1228-1238 (2014).
6. Wang X*, et al.* In Vivo Evidence for ATPase-Dependent DNA Translocation by the Bacillus subtilis SMC Condensin Complex. *Mol Cell* **71**, 841-847 e845 (2018).
7. Meeske AJ*, et al.* MurJ and a novel lipid II flippase are required for cell wall biogenesis in Bacillus subtilis. *Proc Natl Acad Sci U S A* **112**, 6437-6442 (2015).
8. Wagner JK, Marquis KA, Rudner DZ. SirA enforces diploidy by inhibiting the replication initiator DnaA during spore formation in. *Mol Microbiol* **73**, 963-974 (2009).
9. Brandao HB, Ren ZQ, Karaboja X, Mirny LA, Wang XD. DNA-loop-extruding SMC complexes can traverse one another in vivo. *Nat Struct Mol Biol* **28**, 642-+ (2021).
10. Wilhelm L*, et al.* SMC condensin entraps chromosomal DNA by an ATP hydrolysis dependent loading mechanism in Bacillus subtilis. *Elife* **4**, (2015).
11. Karaboja X, Ren Z, Brandão HB, Paul P, Rudner DZ, Wang X. XerD unloads bacterial SMC complexes at the replication terminus. *Mol Cell* **81**, 756-766.e758 (2021).
12. Brandao HB, Paul P, van den Berg AA, Rudner DZ, Wang X, Mirny LA. RNA polymerases as moving barriers to condensin loop extrusion. *Proc Natl Acad Sci U S A* **116**, 20489-20499 (2019).
13. Banigan EJ, van den Berg AA, Brandao HB, Marko JF, Mirny LA. Chromosome organization by one-sided and two-sided loop extrusion. *Elife* **9**, (2020).
14. Le TB, Imakaev MV, Mirny LA, Laub MT. High-resolution mapping of the spatial organization of a bacterial chromosome. *Science* **342**, 731-734 (2013).
