# Supplementary material for: Replisomes restrict SMC translocation in vivo
Source: Nat Commun. 2025 Aug 4;16:7151. doi: 10.1038/s41467-025-62596-y (PMC12322038; doi:10.1038/s41467-025-62596-y)
Supplement: Supplementary file 2 — Description of Additional Supplementary Files [file 41467_2025_62596_MOESM2_ESM.pdf]

Description of Additional Supplementary Files

File name: Supplementary Data 1

Description: Next-generation-sequencing data used in this study

File name: Supplementary Movie 1

Description: SMC translocation is affected by moving replisomes. Related to Fig. 2. Time-course Hi-C maps of a strain containing a single parS site at -27° (BWX5230) after synchronous replication. Samples were taken at 0, 10, 15, 20, 25, 30, 35, 40 min after replication initiation. The left panel shows the 0 min time point. The right panel shows the time course. The positions of parS and replisomes are labeled with black and red dashed lines, respectively. Static images can be found in Figure 2a.

File name: Supplementary Movie 2

Description: SMC translocation is affected by the two-sided head-to-tail collision with the replisome. Related to Fig. 4. Time-course Hi-C maps of a strain containing a single parS site at -1° (BWX4310) after induced SMC loading. Samples were taken at 0, 15, 20, 25 and 30 min after SMC loading. The left panel shows a control experiment with no SMC-replisome collisions, in which replisome is far ahead of SMC. Static images can be found in Figure 4a. The right panel shows DNA juxtaposition when replisomes were stalled by HPUra. Static images can be found in Figure 4b. The positions of parS, moving replisomes, and stalled replisomes are labeled with black dashed lines, red dashed lines and red solid lines, respectively.

File name: Supplementary Movie 3

Description: SMC translocation is affected by the one-sided head-to-tail collision with the replisome. Related to Fig. 5. Time-course Hi-C maps of a strain containing a single parS site at -59° (BWX5297) after induced SMC loading. Samples were taken at 0, 15, 20, and 25 min after SMC loading. The left panel shows a control experiment with no SMC-replisome collisions. Static images can be found in Figure 5a. The right panel shows DNA juxtaposition when replisomes were stalled by HPUra. Static images can be found in Figure 5b. The positions of parS and stalled replisomes are labeled with black dashed lines and red solid lines, respectively.

File name: Supplementary Movie 4

Description: SMC translocation is affected by moving replisomes. Related to Fig. 7. Time-course Hi-C maps of a strain containing a single parS site at -59° after synchronous replication. Samples were taken at 0, 10, 15, 20, 25, 30, 35, 40 min after replication initiation. The left panel (BWX5529) has continuous SMC loading. Static images can be found in Figure 7b. The right panel (BWX5297) has induced SMC loading at the onset of replication initiation. Static images can be found in Figure 7a. The positions of parS and moving replisomes are labeled with black dashed lines and red dashed lines, respectively.
