# Supplementary material for: Replisomes restrict SMC translocation in vivo
Source: Nat Commun. 2025 Aug 4;16:7151. doi: 10.1038/s41467-025-62596-y (PMC12322038; doi:10.1038/s41467-025-62596-y)
Supplement: Supplementary file 8 — Reporting Summary [file 41467_2025_62596_MOESM8_ESM.pdf]

nature portfolio

Corresponding author(s): Xindan Wang

Last updated by author(s): Jul 7, 2025

Reporting Summary

Nature Portfolio wishes to improve the reproducibility of the work that we publish. This form provides structure for consistency and transparency in reporting. For further information on Nature Portfolio policies, see our Editorial Policies and the Editorial Policy Checklist.

Statistics

For all statistical analyses, confirm that the following items are present in the figure legend, table legend, main text, or Methods section.

n/a	Confirmed	
☐	☒	The exact sample size (*n*) for each experimental group/condition, given as a discrete number and unit of measurement
☒	☐	A statement on whether measurements were taken from distinct samples or whether the same sample was measured repeatedly
☒	☐	The statistical test(s) used AND whether they are one- or two-sided<br>*Only common tests should be described solely by name; describe more complex techniques in the Methods section.*
☒	☐	A description of all covariates tested
☒	☐	A description of any assumptions or corrections, such as tests of normality and adjustment for multiple comparisons
☒	☐	A full description of the statistical parameters including central tendency (e.g. means) or other basic estimates (e.g. regression coefficient) AND variation (e.g. standard deviation) or associated estimates of uncertainty (e.g. confidence intervals)
☒	☐	For null hypothesis testing, the test statistic (e.g. *F*, *t*, *r*) with confidence intervals, effect sizes, degrees of freedom and *P* value noted<br>*Give P values as exact values whenever suitable.*
☒	☐	For Bayesian analysis, information on the choice of priors and Markov chain Monte Carlo settings
☒	☐	For hierarchical and complex designs, identification of the appropriate level for tests and full reporting of outcomes
☒	☐	Estimates of effect sizes (e.g. Cohen's *d*, Pearson's *r*), indicating how they were calculated

Our web collection on statistics for biologists contains articles on many of the points above.

Software and code

Policy information about availability of computer code

Data collection	Microscopy data were collected using Nikon Elements 5.11.00 (Build 1346)
Data analysis	Microscopy data are analyzed using Nikon Elements 5.11.00 (Build 1346) 64bit. HiC results were maped using hiclib. ChIP and WGS were mapped using CLC Genomics Workbench (CLC Bio, QIAGEN). Custom algorithms for generating Hi-C and ChIP-seq simulations are deposited at: https://github.com/hbbrandao/bacterialSMCtrajectories

For manuscripts utilizing custom algorithms or software that are central to the research but not yet described in published literature, software must be made available to editors and reviewers. We strongly encourage code deposition in a community repository (e.g. GitHub). See the Nature Portfolio guidelines for submitting code & software for further information.

Data

Policy information about availability of data

All manuscripts must include a data availability statement. This statement should provide the following information, where applicable:

- Accession codes, unique identifiers, or web links for publicly available datasets
- A description of any restrictions on data availability
- For clinical datasets or third party data, please ensure that the statement adheres to our policy

The Hi-C, ChIP-seq and WGS data generated in this study have been deposited in the NCBI Gene Expression Omnibus database under accession code GSE282455

[https://www.ncbi.nlm.nih.gov/geo/query/acc.cgi?acc=GSE282455]. Unprocessed microscopy images are available at Mendeley data (https://data.mendeley.com/datasets/7hrspzt89y/1). Source data are provided with this paper.

Research involving human participants, their data, or biological material

Policy information about studies with human participants or human data. See also policy information about sex, gender (identity/presentation), and sexual orientation and race, ethnicity and racism.

Reporting on sex and gender	N/A
Reporting on race, ethnicity, or other socially relevant groupings	N/A
Population characteristics	N/A
Recruitment	N/A
Ethics oversight	N/A

Note that full information on the approval of the study protocol must also be provided in the manuscript.

Field-specific reporting

Please select the one below that is the best fit for your research. If you are not sure, read the appropriate sections before making your selection.

☒ Life sciences ☐ Behavioural & social sciences ☐ Ecological, evolutionary & environmental sciences

For a reference copy of the document with all sections, see nature.com/documents/nr-reporting-summary-flat.pdf

Life sciences study design

All studies must disclose on these points even when the disclosure is negative.

Sample size	Microscopy images were randomly collected to obtain sufficient number of cells for each dataset. Over 500 cells were analyzed for each time point. This sample size was chosen because 500 is considered sufficient for quantitative analysis.
Data exclusions	No data were excluded. Microscopy samples were allocated into experimental groups based on the number of fluorescence foci in each cell.
Replication	Two independent experiments were performed and produced similar results.
Randomization	Microscopy images were randomly collected.
Blinding	Blinding is not relevant to our study. We are studying bacteria cells. All the cells were analyzed. No data were excluded.

Reporting for specific materials, systems and methods

We require information from authors about some types of materials, experimental systems and methods used in many studies. Here, indicate whether each material, system or method listed is relevant to your study. If you are not sure if a list item applies to your research, read the appropriate section before selecting a response.

Materials & experimental systems

n/a	Involved in the study	
☐	☒	Antibodies
☒	☐	Eukaryotic cell lines
☒	☐	Palaeontology and archaeology
☒	☐	Animals and other organisms
☒	☐	Clinical data
☒	☐	Dual use research of concern
☒	☐	Plants

Methods

n/a	Involved in the study	
☐	☒	ChIP-seq
☒	☐	Flow cytometry
☒	☐	MRI-based neuroimaging

Antibodies

Antibodies used	For ChIP-seq study, 4ul of anti-SMC antibodies were used for each sample. These antibodies were previously published in PMID:

Antibodies used

12421306; they are not commercially available with supplier name, catalog number, clone name or lot number. The antibodies were requested from previously published sources as cited (PMID: 12421306).

Validation

The primary antibodies were validated in Bacillus subtilis using by Western blotting in the sources as cited, and in multiple of our previous studies. We did not re-validate these antibodies in this study.

Plants

Seed stocks

N/A

Novel plant genotypes

N/A

Authentication

N/A

ChIP-seq

Data deposition

☒ Confirm that both raw and final processed data have been deposited in a public database such as GEO.

☒ Confirm that you have deposited or provided access to graph files (e.g. BED files) for the called peaks.

Data access links
May remain private before publication.

NCBI Gene Expression Omnibus GSE282455 [https://www.ncbi.nlm.nih.gov/geo/query/acc.cgi?acc=GSE282455].

Files in database submission

GSM8643504 WGS_PY79_exp
GSM8643505 WGS_BWX3212_exp
GSM8643506 WGS_BWX3381_exp
GSM8643507 WGS_BWX5297_T0
GSM8643508 WGS_BWX5297_30C_IPTG10min_T10
GSM8643509 WGS_BWX5297_30C_IPTG15min_T15
GSM8643510 WGS_BWX5297_42C_IPTG20min_T20
GSM8643511 WGS_BWX5297_42C_IPTG25min_T25
GSM8643512 WGS_BWX5297_42C_IPTG30min_T30
GSM8643513 WGS_BWX5297_42C_IPTG35min_T35
GSM8643514 WGS_BWX5297_42C_IPTG40min_T40
GSM8643515 WGS_BWX5297_42C_IPTG45min_T45
GSM8643516 WGS_BWX5297_42C_IPTG50min_T50
GSM8643517 WGS_BWX5297_30C_T10
GSM8643518 WGS_BWX5297_30C_T15
GSM8643519 WGS_BWX5297_42C_T20
GSM8643520 WGS_BWX5297_42C_T25
GSM8643521 WGS_BWX5297_42C_T30
GSM8643522 WGS_BWX5297_42C_T35
GSM8643523 WGS_BWX5297_42C_T40
GSM8643524 WGS_BWX5297_42C_T45
GSM8643525 WGS_BWX5297_42C_T50
GSM8643526 WGS_BWX5230_T0
GSM8643527 WGS_BWX5230_30C_T10
GSM8643528 WGS_BWX5230_30C_T15
GSM8643529 WGS_BWX5230_42C_T20
GSM8643530 WGS_BWX5230_42C_T25
GSM8643531 WGS_BWX5230_42C_T30
GSM8643532 WGS_BWX5230_42C_T35
GSM8643533 WGS_BWX5230_42C_T40
GSM8643534 WGS_BWX4310_exp
GSM8643535 WGS_BWX4310_T0
GSM8643536 WGS_BWX4310_30C_T15
GSM8643537 WGS_BWX4310_42C_IPTG15min_T30
GSM8643538 WGS_BWX4310_42C_IPTG30min_T45
GSM8643539 WGS_BWX4310_42C_HPUraIPTG15min_T30
GSM8643540 WGS_BWX4310_42C_HPUraIPTG30min_T45
GSM8643541 WGS_BWX4310_42C_IPTG20min_T35
GSM8643542 WGS_BWX4310_42C_IPTG25min_T40
GSM8643543 WGS_BWX4310_42C_HPUraIPTG20min_T35
GSM8643544 WGS_BWX4310_42C_HPUraIPTG25min_T40
GSM8643545 WGS_BWX5297_42C_noreplisome_IPTG15min_T15

GSM8643546 WGS_BWX5297_42C_noreplisome_IPTG20min_T20
GSM8643547 WGS_BWX5297_42C_noreplisome_IPTG25min_T25
GSM8643548 WGS_BWX5297_42C_HPUraIPTG15min_T40
GSM8643549 WGS_BWX5297_42C_HPUraIPTG20min_T45
GSM8643550 WGS_BWX5297_42C_HPUraIPTG25min_T50
GSM8643551 WGS_BWX5297_42C_IPTG20min_HPUra5min_T20
GSM8643552 WGS_BWX5297_42C_IPTG25min_HPUra10min_T25
GSM8643553 WGS_BWX5529_T0
GSM8643554 WGS_BWX5529_30C_T10
GSM8643555 WGS_BWX5529_30C_T15
GSM8643556 WGS_BWX5529_42C_T20
GSM8643557 WGS_BWX5529_42C_T25
GSM8643558 WGS_BWX5529_42C_T30
GSM8643559 WGS_BWX5529_42C_T35
GSM8643560 WGS_BWX5529_42C_T40
GSM8643561 WGS_BWX4504_42C_IPTG1h
GSM8643562 ChIP_anti_SMC_BWX5297_T0
GSM8643563 ChIP_anti_SMC_BWX5297_30C_IPTG10min_T10
GSM8643564 ChIP_anti_SMC_BWX5297_30C_IPTG15min_T15
GSM8643565 ChIP_anti_SMC_BWX5297_42C_IPTG20min_T20
GSM8643566 ChIP_anti_SMC_BWX5297_42C_IPTG25min_T25
GSM8643567 ChIP_anti_SMC_BWX5297_42C_IPTG30min_T30
GSM8643568 ChIP_anti_SMC_BWX5297_42C_IPTG35min_T35
GSM8643569 ChIP_anti_SMC_BWX5297_42C_IPTG40min_T40
GSM8643570 ChIP_anti_SMC_BWX5297_42C_IPTG45min_T45
GSM8643571 ChIP_anti_SMC_BWX5297_42C_IPTG50min_T50
GSM8643572 ChIP_anti_SMC_BWX5297_42C_noreplisome_IPTG25min_T25
GSM8643573 ChIP_anti_SMC_BWX5297_42C_HPUraIPTG25min_T50
GSM8643574 ChIP_anti_SMC_BWX5297_42C_IPTG25min_HPUra10min_T25
GSM8643575 ChIP_anti_SMC_BWX4504_42C_IPTG1h
GSM8643576 ChIP_anti_SMC_BWX5297_42C_IPTG20min_HPUra5min_T20
GSM8643577 HiC_BWX5230_T0
GSM8643578 HiC_BWX5230_30C_T10
GSM8643579 HiC_BWX5230_30C_T15
GSM8643580 HiC_BWX5230_42C_T20
GSM8643581 HiC_BWX5230_42C_T25
GSM8643582 HiC_BWX5230_42C_T30
GSM8643583 HiC_BWX5230_42C_T35
GSM8643584 HiC_BWX5230_42C_T40
GSM8643585 HiC_BWX4310_42C_IPTG15min_T30
GSM8643586 HiC_BWX4310_42C_IPTG20min_T35
GSM8643587 HiC_BWX4310_42C_IPTG25min_T40
GSM8643588 HiC_BWX4310_42C_IPTG30min_T45
GSM8643589 HiC_BWX4310_42C_HPUraIPTG15min_T30
GSM8643590 HiC_BWX4310_42C_HPUraIPTG20min_T35
GSM8643591 HiC_BWX4310_42C_HPUraIPTG25min_T40
GSM8643592 HiC_BWX4310_42C_HPUraIPTG30min_T45
GSM8643593 HiC_BWX5297_T0
GSM8643594 HiC_BWX5297_42C_noreplisome_IPTG15min_T15
GSM8643595 HiC_BWX5297_42C_noreplisome_IPTG20min_T20
GSM8643596 HiC_BWX5297_42C_noreplisome_IPTG25min_T25
GSM8643597 HiC_BWX5297_42C_HPUraIPTG15min_T40
GSM8643598 HiC_BWX5297_42C_HPUraIPTG20min_T45
GSM8643599 HiC_BWX5297_42C_HPUraIPTG25min_T50
GSM8643600 HiC_BWX5297_30C_IPTG15min_T15
GSM8643601 HiC_BWX5297_42C_IPTG20min_HPUra5min_T20
GSM8643602 HiC_BWX5297_42C_IPTG25min_HPUra10min_T25
GSM8643603 HiC_BWX5297_30C_IPTG10min_T10
GSM8643604 HiC_BWX5297_42C_IPTG20min_T20
GSM8643605 HiC_BWX5297_42C_IPTG25min_T25
GSM8643606 HiC_BWX5297_42C_IPTG30min_T30
GSM8643607 HiC_BWX5297_42C_IPTG35min_T35
GSM8643608 HiC_BWX5297_42C_IPTG40min_T40
GSM8643609 HiC_BWX5529_T0
GSM8643610 HiC_BWX5529_30C_T10
GSM8643611 HiC_BWX5529_30C_T15
GSM8643612 HiC_BWX5529_42C_T20
GSM8643613 HiC_BWX5529_42C_T25
GSM8643614 HiC_BWX5529_42C_T30
GSM8643615 HiC_BWX5529_42C_T35
GSM8643616 HiC_BWX5529_42C_T40
GSM8643617 Hi-C_BWX4310_42C_IPTG15min
GSM8643618 Hi-C_BWX4310_42C_IPTG15min_HPUra15min
GSM8643619 Hi-C_BWX4310_42C_IPTG30min
GSM8972109 WGS_BWX4310_42C_HPUraIPTG30min_T45_rep2
GSM8972110 WGS_BWX5297_42C10m_T0

GSM8972111 ChIP_anti_SMC_BWX4310_42C_HPUraIPTG15min_T30
GSM8972112 ChIP_anti_SMC_BWX4310_42C_HPUraIPTG20min_T35
GSM8972113 ChIP_anti_SMC_BWX4310_42C_HPUraIPTG25min_T40
GSM8972114 ChIP_anti_SMC_BWX4310_42C_HPUraIPTG30min_T45
GSM8972115 HiC_BWX5297_42C10m_T0
GSM9087550 WGS_input_BWX3212
GSM9087551 ChIP_anti_SMC_BWX3212

Genome browser session (e.g. UCSC)	https://indiana-my.sharepoint.com/:f:/g/personal/xindan_iu_edu/EqH_nMxJi8JJjM4OBAf3OT8BiBVZvBDZMNRAzzpXGDhkjA?e=TBsxoE

Methodology

Replicates	The data presented are shown in 1 replicate.
Sequencing depth	ChIP-seq reads were sequenced using paired-end sequencing with 39nt per read. Over two million raw reads and uniquely mapped reads were generated for each sample.
Antibodies	anti-SMC antibodies were used for ChIP-seq. They were requested from previously published sources as cited.
Peak calling parameters	Not applicable. Genome-wide distribution of reads were plotted. Peak calling was not performed.
Data quality	This study did not involve peak calling. Genome-wide distribution of reads were plotted. We made sure that every sample had greater than two million reads.
Software	Reads were mapped to the genome using CLC genomics workbench (Qiagen). The data were plotted using R.
